# An Inducible BRCA1 Expression System with In Vivo Applicability Uncovers Activity of the Combination of ATR and PARP Inhibitors to Overcome Therapy Resistance

**DOI:** 10.3390/cancers18020309

**Published:** 2026-01-20

**Authors:** Elsa Irving, Alaide Morcavallo, Jekaterina Vohhodina-Tretjakova, Paul W. G. Wijnhoven, Anna L. Beckett, Michael P. Jacques, Rachel S. Evans, Jennifer I. Moss, Anna D. Staniszewska, Josep V. Forment

**Affiliations:** Bioscience, Oncology Targeted Discovery, Oncology R&D, AstraZeneca, Cambridge CB2 0AA, UK; elsa.irving1@astrazeneca.com (E.I.); alaide.morcavallo@astrazeneca.com (A.M.); jekaterina.vohhodina@astrazeneca.com (J.V.-T.); paulus.wijnhoven@astrazeneca.com (P.W.G.W.); annalouise.beckett@astrazeneca.com (A.L.B.); michaeljacques78@gmail.com (M.P.J.); rachel.evans3@astrazeneca.com (R.S.E.); jennifer.hare@astrazeneca.com (J.I.M.); anna.staniszewska@astrazeneca.com (A.D.S.)

**Keywords:** BRCA mutant, BRCA hypomorph, PARP inhibitors, resistance, breast cancer, combination therapy

## Abstract

Background: Poly(ADP-ribose) polymerase inhibitors (PARPi) have transformed cancer therapy for patients harbouring homologous recombination repair (HRR) deficiencies, notably *BRCA1/2* mutations. However, resistance to PARPi remains a clinical challenge, with restoration of BRCA1 function via hypomorphic variants representing an understudied scenario. Methods: Here, we engineered a doxycycline-inducible BRCA1 expression system in the *BRCA1*-mutant, triple-negative breast cancer cell line MDAMB436, permitting controlled analysis of functionally distinct BRCA1 hypomorphs in vitro and in vivo. Results: Among multiple BRCA1 variants generated—including RING, coiled-coil, and BRCT domain mutants—only overexpression of the ∆exon11 hypomorph robustly conferred resistance to olaparib and carboplatin, with drug sensitivity correlating to ∆exon11 expression levels. While ∆exon11 BRCA1 mediated HRR restoration, its efficiency was consistently lower than full-length BRCA1, as measured by RAD51 foci formation and interaction with repair partners such as PALB2. In vivo, tumours expressing Δexon11 BRCA1 exhibited only partial resistance to olaparib compared to those expressing full-length BRCA1. Importantly, the combination of olaparib and the ATR inhibitor, ceralasertib, overcame ∆exon11-mediated resistance, impairing RAD51 foci formation in ∆exon11-expressing cells. Conclusions: Our findings identify a dose-dependent, hypomorphic HRR restoration by ∆exon11 BRCA1, help explain the variable resistance observed in BRCA1-mutant pre-clinical models expressing this hypomorph, and propose ATR inhibition in combination with PARPi as a clinical strategy to counteract therapeutic resistance mediated by ∆exon11 BRCA1 hypomorphs.

## Simple Summary

Poly(ADP-ribose) polymerase inhibitors (PARPi) benefit cancers with homologous recombination repair (HRR) defects, yet resistance emerges. Using a doxycycline-inducible system in BRCA1-mutant MDA-MB-436 cells, we tested different BRCA1 hypomorphs. Among all the variants tested, only the ∆exon11 BRCA1 isoform conferred robust, dose-dependent resistance to olaparib and carboplatin. BRCA1 ∆exon11 partially restored HRR—less efficiently than full-length BRCA1—based on RAD51 foci and PALB2 interaction. In vivo, BRCA1 ∆exon11 yielded partial resistance to olaparib. Notably, combining olaparib with the ATR inhibitor ceralasertib abrogated resistance by suppressing RAD51 foci, supporting PARPi–ATR inhibitor combinations to overcome BRCA1 ∆exon11-mediated resistance.

## 1. Introduction

Inhibitors of poly(ADP ribose) polymerases (PARPi) have revolutionised the treatment landscape for cancer patients with tumours harbouring mutations and/or genetic signatures of homologous recombination repair (HRR) defects, including those affecting the *BRCA1* or *BRCA2* tumour suppressor genes. Accordingly, several PARPis are now approved as monotherapies and in combinations for patients with tumours carrying such alterations in different disease settings. However, PARPi resistance arises, with several molecular mechanisms driving resistance having been described in clinical and pre-clinical settings [1]. Although several approaches have been considered to overcome PARPi resistance, including combinations with inhibitors targeting the DNA-damage response (DDR) (e.g., ATR or WEE1 inhibitors), the molecular understanding to tailor the right combination approach to specific biomarker-selected patient populations needs further investigation [2,3].

**Figure 1:**
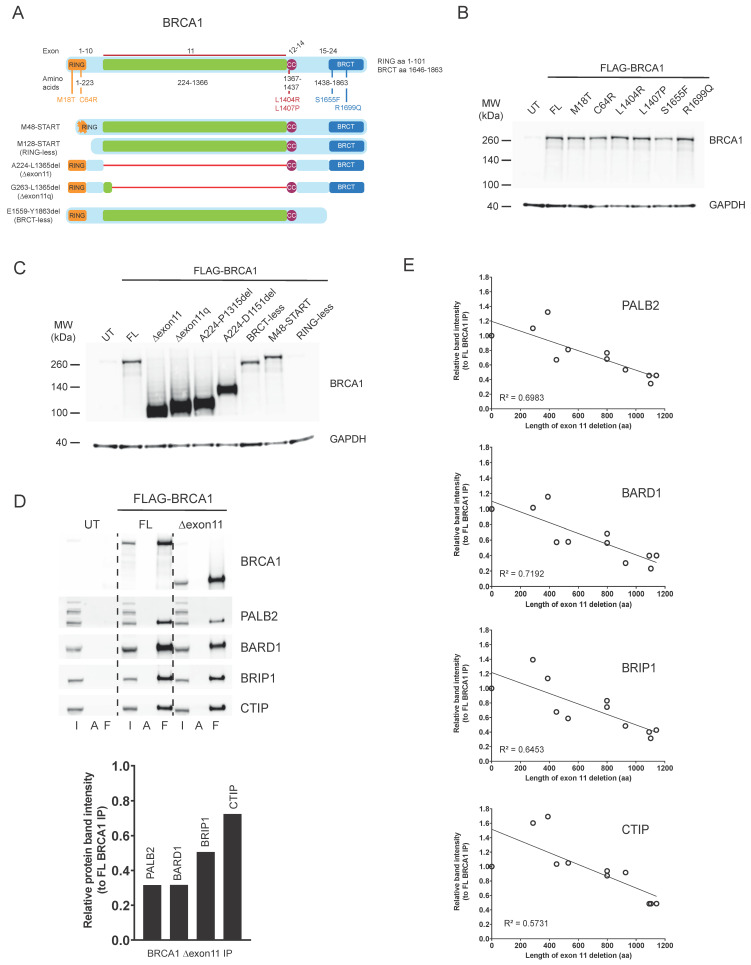
(**A**) Schematic of the BRCA1 protein and its functional domains, including the single amino acid changes analysed in this study (top). Deletions representing hypomorphic variants affecting different domains are depicted at the bottom. (**B**) Western blot showing expression in HEK293T cells of FLAG-tagged BRCA1 constructs containing the full length (FL) protein or the different amino acid changes assessed in this study. GAPDH was used as a loading control. UT: untransfected. (**C**) Western blot showing expression in HEK293T cells of FLAG-tagged BRCA1 constructs containing the full length (FL) protein or the different deletion mutants assessed in this study. GAPDH was used as a loading control. UT: untransfected. (**D**) (**Top panel**)*:* co-immunoprecipitation experiments in HEK293T cells with full-length (FL) or ∆exon11 FLAG-BRCA1. (**Bottom panel**)*:* relative pull-down efficiency of BRCA1 interactors with the FLAG-BRCA1 ∆exon11 construct, compared to FL protein. I: input; A: pull down with protein A beads; F: pull down with FLAG beads. (**E**) Relative pull-down efficiency of BRCA1 interactors with an allelic series of FLAG-BRCA1 deletions inside exon 11, compared to FL protein. R-squared values were derived from simple linear regression analyses. The original western blot figures can be found in Supplementary File S1.

BRCA1 is a multi-functional protein, with several domains involved in different aspects of DNA repair and cell cycle regulation (Figure 1A) [4]. The N-terminal RING domain is required for its interaction with BARD1 and is essential for the enzymatic ubiquitin E3 ligase activity of the BRCA1-BARD1 complex [5]. The central region of the protein is encoded in a single exon (exon 11), and it is a largely unstructured domain that contains a binding region for the RAD51 recombinase, among other features [6]. The coiled-coil domain that sits immediately downstream of the exon 11 sequence is required for the interaction of BRCA1 with the PALB2-BRCA2-RAD51 complex, which is essential for the role of BRCA1 in HRR [7]. And the C-terminal BRCT domains of BRCA1 mediate protein–protein interactions with BRIP1, CTIP, and the BRCA1-A complex, among others [4]. Interestingly, pathogenic mutations in BRCA1 span the entire length of the protein, suggesting that all domains could be important for its function as a tumour suppressor protein [8]. However, whether all its functions are required to confer resistance to platinum drugs or PARPi is a matter of debate. As such, expression of hypomorphic versions of the BRCA1 protein has been associated with resistance to platinum drugs and PARPi in pre-clinical models. Hypomorphs lacking part or the entire N-terminal RING domain of BRCA1 have been described in vitro, in vivo, and in patient-derived xenograft (PDX) models, and have been postulated to be produced by alternative transcriptional START sites downstream of the sequence encoding the RING domain in the *BRCA1* transcript [9,10,11,12]. Similarly, alternative splicing isoforms of *BRCA1* have been described that bypass most or the entire exon 11 sequence (BRCA1 ∆11 or ∆11q) [13], which are invariably detected in vitro [14] and in PDX models [15] where the original *BRCA1* pathogenic mutation lies within exon 11 of the gene. Hypomorphs lacking one or both BRCT domains of BRCA1 have also been described in vitro and in PDX models, and their expression has been linked to chaperone-mediated stabilisation and genomic rearrangements [16,17], and associated with resistance to treatment [12].

Modelling the effect of BRCA1 hypomorph expression in pre-clinical models has been difficult due to two major problems: the detrimental effect of clinically relevant point mutations on BRCA1 protein levels [7,18], and the lack of in vivo inducible expression systems to assess the impact of varying levels of hypomorph expression on therapy responses. To circumvent these issues, we describe here the development of a doxycycline-inducible BRCA1 expression system in the clinically relevant BRCA1 mutant, triple-negative breast cancer cell line MDAMB436. We further demonstrate the utility of this system by describing the requirement of substantial levels of overexpression of the BRCA1 hypomorph lacking the entire exon 11 of the gene (BRCA1 ∆exon11) to generate resistance in vivo to PARPi, and by identifying the combination of PARPi with inhibitors of the DDR kinase ATR as a way to overcome such resistance.

## 2. Materials and Methods

### 2.1. Cell Lines and Chemicals

SUM149PT (300609) was sourced from Cyton and grown in HAM’s F12 (Gibco, Waltham, MA, USA) supplemented with 5% foetal bovine serum (FBS) (Sigma, Kanagawa, Japan), 500 ng/mL Hydrocortisone (Gibco), 10 µg/mL human recombinant insulin (Gibco), and 1× GlutaMAX (Gibco). All other cell lines were sourced from ATTC. HEK-293T (CRL-3216), MCF7 (HTB-22), and MDAMB231 were grown in DMEM (Gibco), MDAMB436 and HCC1937 were grown in RPMI 1640 (Sigma), all supplemented with 10% FBS and 1X GlutaMAX. UWB1.289 (CRL-2945) and UWB1.289 + BRCA1 (CRL-2946) were grown in 50% RPMI 1640, 50% M171 supplemented with 3% FBS and 1X Mammary Epithelial Growth Supplement (MEGS) (Thermo Fisher, Waltham, MA, USA). Cells were maintained at 37 °C in 5% CO_2_. Olaparib, carboplatin, ceralasertib, and adavosertib were made by AstraZeneca (Cambridge, UK) and were dosed in DMSO (DMSO-normalised across conditions). Tetracycline and doxycycline were sourced from Thermo Fisher and Sigma, respectively.

### 2.2. Constructs

Plasmids containing the human *BRCA1* (NM_007294) and *RAD51B* (NM_133509) open reading frames (ORFs) were obtained from Genscript (Ohu18572D) and OriGene (RC206457L3), respectively. BRCA1 deletion and mutant constructs were generated using the Q5 site-directed mutagenesis kit (New England Biolabs, Ipswich, MA, USA) and custom primers, sequences confirmed by Sanger sequencing.

### 2.3. Transient Transfection

HEK-293T cells were transfected using a 3.5:1 ratio of FuGENE HD (Promega, Madison, WI, USA) to DNA, following the manufacturer’s instructions. Cells were lysed for protein after 48/72 h, and samples were processed for Western blotting or co-immunoprecipitation.

### 2.4. Lentivirus Generation

HEK-293T cells were transfected with the gene of interest lentiviral expression plasmid, psPAX2 (lentiviral packaging plasmid), and mMD2.G VSV-G (lentiviral envelope plasmid) using Lipofectamine LTX (Thermo Fisher). The media was changed after 6 h. 72 h post-transfection, lentivirus-containing media were harvested, syringe-filtered (0.45 μM), and stored at −80 °C.

For *BRCA1*, prior to lentivirus generation, ORFs were cloned into a tetracycline-regulated lentiviral expression vector from the ViraPower™ HiPerform™ T-REx™ Gateway™ Vector Kit (Thermo Fisher). This kit also contained a control lentiviral vector for overexpression (LacZ plasmid) and a lentiviral vector for constitutive expression of the tetracycline-repressor protein.

### 2.5. Cell Line Generation

MDAMB436 overexpression cell lines were generated by lentiviral transduction. Lentivirus was added to parental cells along with 8 μg/mL polybrene (Millipore, Burlington, MA, USA), and the media were replaced after 24 h. After a further 24/48 h, the antibiotic was added to select for successfully transduced cells. 1 μg/mL puromycin (Gibco) was used to select for cells infected with the RAD51B lentivirus; 500 μg/mL G418 (Thermo Fisher) was used for tetracycline-repressor protein, and 12 μg/mL blasticidin (Thermo Fisher) was used for BRCA1. Cells were then expanded in the presence of an antibiotic and used for the experiments described here as polyclonal populations to avoid clonal selection issues. For the MDAMB426-B-TR + BRCA1 cells, tetracycline/doxycycline was added to the cells to induce *BRCA1* gene expression (1 mg/mL unless otherwise stated).

### 2.6. (Co-)Immunoprecipitation

Cells were lysed for protein in immunoprecipitation lysis buffer (300 mM NaCl, 1 mM EDTA, 20 mM Tris-HCL, 0.5% IGEPAL, 10% glycerol) supplemented with protease inhibitor (Roche, Basel, Switzerland). Protein lysates of equal concentration were incubated with Anti-FLAG (Sigma F2426) or Protein A control (Sigma P6486) affinity gel at 4 °C for at least 4 h (up to overnight). Flow-through lysates were collected, after which beads were washed with lysis buffer (5 min at 4 °C, 3 times), and bound proteins were eluted in NuPAGE^TM^ LDS sample buffer (Invitrogen NP0007, Carlsbad, CA, USA) supplemented with reducing agent (Invitrogen NP0009) by heating to 95 °C for 5 min. Samples were then processed for Western blotting.

### 2.7. Western Blotting

Protein lysates were prepared in Laemmli buffer, RIPA buffer (Thermo Fisher) (supplemented with phosphatase inhibitor, protease inhibitor, 1% Triton X-100, and 50 U/mL benzonase), or were used for co-immunoprecipitation experiments. Lysates were normalised to equal protein concentration and mixed with NuPAGE^TM^ LDS sample buffer (Invitrogen NP0007) supplemented with reducing agent (Invitrogen NP0009), before heating to 95 °C for 10 min. Sodium dodecyl sulfate–polyacrylamide gel electrophoresis was performed by loading samples into NuPAGE™ 4–12% Bis-Tris protein gels and using NuPAGE™ MOPS SDS running buffer (Invitrogen), running at 140 V until the sample buffer reached the bottom of the gel. Wet transfer onto nitrocellulose (Invitrogen) was performed overnight at 30 V, 4 °C, followed by blocking with non-fat milk (5% Marvel in TBS, 0.05% Tween). Membranes were incubated in primary antibodies (Table 1) diluted in blocking buffer at 4 °C overnight, followed by washing in TBS, 0.05% Tween. Next, membranes were incubated in horseradish peroxidase (HRP)-conjugated secondary antibodies diluted in blocking buffer at room temperature for 1 h and washed again. Bands were visualised using ECL reagent (Thermo Fisher) and imaged using the G:BOX system (Syngene, Baltimore, MD, USA) or X-ray film (Amersham Hyperfilm, GE Healthcare, Buckinghamshire, UK). Band intensity was quantified using ImageJ 2.3.0.

### 2.8. Colony Forming Assay

Cells were dosed with olaparib using the HP D300 digital dispenser (Tecan, Männedorf, Switzerland) and cultured for 14 days in the presence of the drug, after which they were fixed and stained with Blue-G-250 Brilliant Blue (Sigma B8522-1EA) reconstituted in 25% methanol and 5% acetic acid. Once dry, plates were imaged using the GelCount system (Oxford Optronix, Adderbury, UK) and colony density was measured using ImageJ. Curves were plotted using GraphPad Prism 10.4.1, and non-linear regression was used to calculate IC50s. Statistical analysis was performed using a One-Way ANOVA with Holm–Sidak post hoc testing.

### 2.9. Survival Assays

Cells were dosed with various compounds as single agents using the HP D300 (Tecan) and cultured for 14 days in the presence of the drugs, after which cell viability was assessed using Celltiter-Glo 2 (Promega). Curves were plotted using GraphPad Prism, and non-linear regression was used to calculate IC50s. Statistical analysis was performed using a One-Way ANOVA with Holm–Sidak post hoc testing.

For combination assays, cells were dosed using the HP Echo 555 (Labcyte, San Jose, CA, USA) and cultured for seven or 10 days in the presence of the drugs, after which cell viability was assessed using Celltiter-Glo or Celltiter-Glo 2 (Promega). Data were analysed using the Genedata Screener platform, including generating HSA scores for combination activity.

### 2.10. Proliferation Assays

Cells were plated in 1 μg/mL doxycycline to induce BRCA1 expression, and confluency was monitored over 7 days using IncuCyte (Sartorius, Göttingen, Germany). Data were plotted using GraphPad Prism, and non-linear regression was used to fit curves.

### 2.11. EdU Immunofluorescence Assay

Cells were seeded into poly-L-lysine (PLL) coated 96-well plates with 1 µg/mL doxycycline (to induce BRCA1 expression). One hour prior to fixation with 4% paraformaldehyde (10 min at room temperature), cells were treated with 10 μM EdU. Cells were permeabilised with PBS containing 0.2% Triton X-100 for 10 min at room temperature, and blocking was performed using 0.5% BSA and 0.2% gelatine from cold-water fish skin (Sigma) in PBS for 1 h at room temperature. An EdU Click-It reaction was then performed by incubating cells in 2 mM CuSO_4_·5H_2_O, 100 mM ascorbate, 5 μM Alexa azide 647 (Thermo Fisher) in HEPES, pH 7, for 20 min at room temperature. Nuclei were labelled using 1 μg/mL DAPI. EdU-positive nuclei were detected, and image analysis was performed using ScanR.

### 2.12. RT-qPCR

RNA was extracted from in vitro cultures using the RNeasy Plus kit (Qiagen, Hilden, Germany) following the manufacturer’s instructions. RNA extraction and purification from tumours was performed using the RNeasy 96 QIAcube kit (QIAGEN, #74171). Next, cDNA was synthesised using the SuperScript IV VILO kit (Thermo Fisher) following the manufacturer’s instructions. RT-qPCR was then performed on the QuantStudio™ 6 Pro (Thermo Fisher) using custom primers (Table 2) designed to amplify exogenous FLAG-tagged *BRCA1* specifically (or *GAPDH* as a housekeeping control) and the PowerTrack™ SYBR Green Master Mix (Thermo Fisher). Data were analysed using the delta delta Cq method and plotted in GraphPad Prism.

### 2.13. Animal Studies

All the in vivo experimental protocols were monitored and approved by AstraZeneca Animal Welfare and Ethical Review Body, in compliance with guidelines specified by the UK Home Office Animals (Scientific Procedures) Act 1986 and AstraZeneca Global Bioethics policy or Institutional Animal Care and Use Committee (IACUC), PREPARE guidelines, and reported in line with the ARRIVE guidelines. The experimental work is outlined in project licence PP3292652, which has gone through the AstraZeneca Ethical Review Process. Five mice per individually vented cage, enriched with corncob bedding, nesting material, and solid plastic enrichment tubes, were maintained in a controlled, specific pathogen-free environment at 20 °C to 25 °C, 40% to 70% humidity, and a 12 h light-to-dark cycle. Mice were allowed access to food and water ad libitum and were euthanized at the appropriate humane endpoint.

To induce *BRCA1* gene expression, doxycycline + sucrose (stock 6.7 mg/mL + 135 mg/mL) was dissolved in drinking water to reach a final concentration of 0.5 mg/mL + 16 mg/mL. Water pouch replacement was performed regularly every 4 days to prevent degradation and contamination.

MDA-MB-436, MDAMB426-B-TR + FL BRCA1 or MDAMB426-B-TR + BRCA1 Δexon11 cells (5 × 10^6^ with 50% Matrigel (Corning, New York, NY, USA)) were implanted subcutaneously into 5–10 weeks old SCID (C.B-17/IcrHsd-Prkdcscid) female mice (Envigo, London, UK). Animal body weight and tumour condition were monitored throughout the study. Tumour length and width were measured using a calliper, and tumour volume (TV) was calculated using the formula volume = (length × width^2^) × π/6. Mice were randomised, using in-house developed software, into treatment groups (mice *n* = 10, or *n* = 8, or *n* = 8, respectively) when mean TV reached approximately 0.2 cm^3^. Group sizes were determined based on historical model data and expected effect size.

Tumour growth inhibition (TGI) from the start of treatment was assessed by comparison of the mean change in TV of the control and treated groups and represented as per cent TGI (when TV ≥ starting TV) or tumour regression (TR, when TV < starting TV). Statistical significance was evaluated using a one-tailed *t*-test. Data are presented as treatment group geomeans, with error bars depicting SEM (calculated with internal software) as per AstraZeneca best practices.

Olaparib was formulated in 10% DMSO, 30% kleptose, and dosed continuously; ceralasertib was formulated in 10% DMSO, 40% propylene glycol, and dosed on a schedule of 3 times/week, BID.

Mice received treatment for a maximum of 42 days following randomization.

### 2.14. Immunofluorescence

Cells were seeded in 96-well plates using media containing 1 μg/mL doxycycline to induce BRCA1 expression. The following day, cells were treated with 0.3 μM ceralasertib for 24 h. Subsequently, cells were exposed to 5 Gy of ionising radiation (IR) using a high-voltage X-ray generator (Faxitron X-Ray Corporation, Tucson, AZ, USA). Four hours post-IR, cells were fixed with 4% paraformaldehyde for 15 min at room temperature (RT), followed by permeabilisation with 1× PBS containing 0.1% Triton X-100 for 10 min at RT. Blocking was performed using 0.5% BSA and 0.2% gelatin from cold-water fish skin (Sigma) in 1× PBS for 1 h at RT.

Cells were then incubated overnight at 4 °C with primary antibodies, followed by incubation at RT with Alexa Fluor-conjugated secondary antibodies and DAPI (Sigma, 1 μg/mL) for 1.5 h (Table 3).

Image acquisition was carried out using an Olympus ScanR microscope with a 40× objective to analyse DNA damage-induced foci. Cell cycle distribution was determined based on DAPI staining intensity, and foci were quantified using the “spots detector module” in cells at the S–G2 phases.

## 3. Results

### 3.1. BRCA1 Hypomorphic Versions Display Different Protein–Protein Interactions

To better understand the importance of the different BRCA1 functions and domains with respect to response to PARPi, we decided to generate an allelic series capturing point mutations and deletions in different BRCA1 regions (Figure 1A). Regarding mutations, we produced M18T and C64R in the RING domain, as well as S1655F and R1699Q in the BRCT domains, as these are well-described germline pathogenic mutations in BRCA1 that have been shown not to result in substantially reduced protein levels when exogenously expressed in human cells [7,18]. In addition, we generated two mutations in the coiled-coil domain (L1404R and L1407P) that have been described to disrupt the interaction between BRCA1 and PALB2, which is essential for HRR [7]. Regarding deletions, we generated two variants with alternative START codon sites that partially (M48 START) or completely (M128 START, which we named RING-less) remove the N-terminal RING domain of BRCA1, and that have been postulated as alternative translation start points to generate RING-defective BRCA1 hypomorphs [10]. We also produced variants lacking the entire exon 11 of the gene (A224-L1365del, which we named ∆exon11) or only retaining its first 40 amino acids (G263-L1365del, which we named ∆exon11q), as these can be produced through alternative splicing of the *BRCA1* transcript [13]. Finally, we also generated a BRCA1 version lacking the C-terminal BRCT domains (E1559-Y1863del, which we named BRCT-less), as similar BRCA1 hypomorphic variants have been linked to PARPi resistance in preclinical models [16,17].

FLAG-tagged constructs with all these variants were produced, and expression was tested upon human cell line transfection. All constructs carrying point mutations were expressed at similar levels to the wild-type, full-length (FL) BRCA1 protein, and over-expressed compared to the endogenous BRCA1 protein levels (Figure 1B). Deletion constructs in the RING (M48 START) and BRCT regions were also expressed at similar levels compared to the FL, while exon 11 deletions resulted in significantly higher protein levels than the FL protein, as previously observed [14,19] (Figure 1C). To assess whether point mutations and deletions affected protein–protein interactions as previously described, we immunoprecipitated the different FLAG-tagged BRCA1 constructs and probed for the RING-interacting protein BARD1, the coiled-coil interacting protein PALB2, and the BRCT-interacting proteins BRIP1 and CTIP [7]. As expected, RING mutants and deletions (M18T, C64R, M48 START, M128 START) all failed to interact with BARD1; the coiled-coil mutants L1404R and L1407P failed to pull down PALB2, and the BRCT mutants and deletion (S1655F, R1699Q, E1559-Y1863del) did not show an interaction with BRIP1 or CTIP (Figure S1A). Despite not being able to detect the BRCA1 RING-less mutant by Western blotting (due to the antibody epitope being in the RING domain; Figure 1C), we did observe it pulling down PALB2, BRIP1, and CTIP, confirming its expression (M128 START pull downs; Figure S1A).

We found that, despite their significantly higher expression, the exon 11 deletions did not seem to co-immunoprecipitate more BRCA1-interacting partners (Figure S1A). To confirm this observation, we adjusted BRCA1 protein concentration levels between FL and ∆exon11 (Figure S1B) and quantified the amount of BRCA1 interactors co-immunoprecipitated. This highlighted that the BRCA1 ∆exon11 protein, despite being able to co-immunoprecipitate all the interactors tested, did so less efficiently than the FL protein, particularly in the case of PALB2 and BARD1 (Figure 1D). To better understand whether there were any specific sequence requirements inside the region encoded by exon 11 to sustain effective protein–protein interactions, we generated an additional allelic series of deletions of different sizes inside exon 11, including two (R762-D1151del and E427-S713del) reported as secondary reversion mutations detected in patients progressing on PARPi treatment (Figure S1C) [20,21]. Importantly, even though all of them co-immunoprecipitated the different interaction partners tested (Figure S1D), there was a clear correlation between the size of the exon 11 deletion and the ability of BRCA1 to perform such interactions (Figure 1E).

### 3.2. Generation of a Doxycycline Inducible BRCA1 Expression System

To better control the effect of the cellular response to PARPi of the different mutations or deletions introduced in BRCA1, we decided to generate a doxycycline-inducible expression system in the triple-negative breast cancer cell line, MDAMB436. We chose this cell line because it carries a hemizygous *BRCA1* mutation (5396 + 1G > A mutation in the splice donor site of exon 20) [22] that results in strongly reduced levels of BRCA1 protein [14] and exquisite sensitivity to PARPi in vitro and in vivo [23]. Interestingly, examination of the MDAMB436 genetic make-up in the Cancer Cell Line Encyclopaedia [24] highlighted that, in addition to the described *BRCA1* mutation, this cell line also carries a homozygous deletion of the HRR gene *RAD51B* (Figure S2A). As RAD51B loss confers a certain degree of PARPi sensitivity [25], we first produced an MDAMB436 derivative cell line with constitutive expression of RAD51B (MDAMB436-B; Figure S2B). We then introduced the tetracycline repressor in both MDAMB436 and MDAMB436-B (Figure S2C) and named these cell lines MDAMB436-TR and MDAMB436-B-TR, respectively. We transduced them with constructs expressing LacZ (as a control) or FL FLAG-tagged BRCA1, and the inducibility of the system was confirmed by western blotting (Figure S2D).

**Figure 2:**
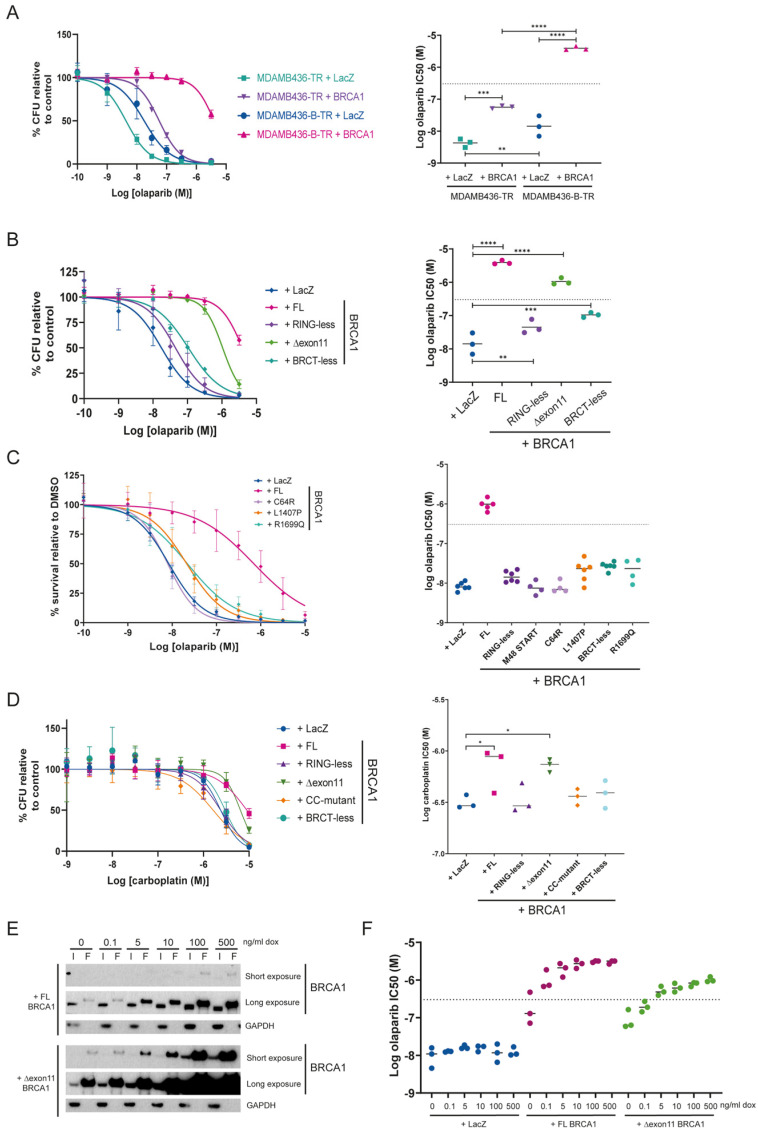
(**A**) (**Left panel**): dose–response curves of olaparib in colony formation assays in tetracycline-repressor (TR) expressing MDAMB436 cells with or without RAD51B complementation (MDAMB436-B-TR and MDAMB436-TR, respectively) and with (+BRCA1) or without (+LacZ) BRCA1 complementation. (**Right panel**): Logarithmic half-maximal inhibitory concentration (LogIC50) of olaparib for each cell line. (**B**) (**Left panel**)*:* dose–response curves of olaparib in colony formation assays in MDAMB436-B-TR cells expressing full-length BRCA1 (+FL), no BRCA1 (+LacZ), or different BRCA1 hypomorphs (+RING-less, +∆exon11, +BRCT-less). (**Right panel**): Logarithmic half-maximal inhibitory concentration (LogIC50) of olaparib for each cell line. (**C**) (**Left panel**): dose–response curves of olaparib in survival assays in MDAMB436-B-TR cells expressing full-length BRCA1 (+FL), no BRCA1 (+LacZ), or different BRCA1 hypomorphs (+C64R, +L1407P, +R1699Q). (**Right panel**): Logarithmic half-maximal inhibitory concentration (LogIC50) of olaparib for each cell line. Data for BRCA1 hypomorphs with deletions in the RING (RING-less, M48 START) or BRCT (BRCT-less) domains are included for comparative purposes. (**D**) (**Left panel**): dose–response curves of carboplatin in survival assays in MDAMB436-B-TR cells expressing full-length BRCA1 (+FL), no BRCA1 (+LacZ) or different BRCA1 hypomorphs (+RING-less, +∆exon11, +BRCT-less, +CC-mutant). (**Right panel**): Logarithmic half-maximal inhibitory concentration (LogIC50) of carboplatin for each cell line. (**E**) Western blot of immunoprecipitation experiments in MDAMB436-B-TR cells expressing full-length (+FL) or ∆exon11 BRCA1 and exposed to different doxycycline doses. GAPDH was used as a loading control. I = input; F = FLAG immunoprecipitation. (**F**) Logarithmic half-maximal inhibitory concentration (LogIC50) of olaparib at the different doses of doxycycline used. All data are from at least 3 biological replicates. Statistical analysis performed using One-Way ANOVA with Holm–Sidak multiple comparisons, * *p* < 0.05, ** *p* < 0.01, *** *p* < 0.001, **** *p* < 0.0001. CFU = colony-forming units. Dotted lines in the olaparib IC50 graphs represent the minimal free concentration of olaparib (approximately 300 nM) in the plasma of patients on the established monotherapy dose of 300 mg twice daily. The original western blot figures can be found in Supplementary File S1.

To understand the importance of RAD51B expression in the MDAMB436 complementation system, we assessed olaparib sensitivity in MDAMB436-TR (lacking RAD51B expression) and MDAMB436-B-TR (expressing RAD51B) cells complemented with FL BRCA1 or control (LacZ) in the presence of doxycycline induction. As shown in Figure 2A, cells expressing RAD51B but not BRCA1 (MDAMB436-B-TR + LacZ) were only marginally more resistant to the PARPi, olaparib (half-maximal inhibitory concentration (IC50) of 14.2 nM), than their *RAD51B* null counterpart (MDAMB436-TR + LacZ cells, IC50 = 4.3 nM), as expected given the dominant effect of BRCA1 loss in driving sensitivity to PARPi. Perhaps surprisingly, however, BRCA1 expression in the absence of RAD51B complementation (MDAMB436-TR + BRCA1 cells) only resulted in mild but significantly increased resistance to olaparib (IC50 = 56.2 nM) when compared to their BRCA1 mutant counterpart (MDAMB436-TR + LacZ cells; IC50 = 4.3 nM). Substantial resistance to olaparib was only achieved by expression of both RAD51B and BRCA1 (MDAMB436-B-TR + BRCA1; IC50 = 3899 nM). The IC50 value of olaparib in the MDAMB436-B-TR + BRCA1 system is consistent with those in HRR proficient cell lines [23], suggesting that our complementation system recapitulates restoration of BRCA1 expression. Consequently, all further experiments were performed in the MDAMB436-B-TR background (hereafter referred to as BTR cells).

As we wanted to understand how stringent repression of BRCA1 expression was in BTR cells, we tested their sensitivity to olaparib in the presence or absence of doxycycline induction. We observed a mild increase in resistance to olaparib in BTR cells complemented with FL BRCA1, even in the absence of doxycycline induction (BTR + BRCA1-dox; IC50 = 109 nM) when compared to non-BRCA1 complemented cells (BTR + LacZ + dox; IC50 = 17 nM) (Figure S2E). Inspection of BRCA1 expression by western blotting detected low levels of the FL protein even in the absence of doxycycline induction, potentially explaining the observed increased resistance to olaparib (Figure S2F). As expected, doxycycline-induced *BRCA1* expression resulted in significantly increased BRCA1 protein levels (Figure S2F) and increased resistance to olaparib (BTR + BRCA1 + dox; IC50 = 3962 nM) (Figure S2E).

Taken together, these results show functionality of the doxycycline-inducible BRCA1 complementation system, and the requirement of both RAD51B and BRCA1 complementation in MDAMB436 cells to generate resistance to olaparib to levels comparable to cell lines with no HRR deficiency [25].

### 3.3. Expression of the BRCA1 ∆exon11 Hypomorph Generates Resistance to Olaparib and Carboplatin In Vitro

In addition to the FL BRCA1, BTR cell lines expressing RING mutants (RING-less and C64R), the exon 11 deletion (∆exon11), a coiled-coil mutation (L1407P), or BRCT mutants (BRCT-less and R1699Q) of BRCA1 were also produced (Figure S2F). Interestingly, while expression of RING-less (IC50 = 45 nM) or BRCT-less BRCA1 (IC50 = 106 nM) resulted in a mild increase in olaparib resistance compared to the LacZ control (IC50 = 17 nM), expression of the ∆exon11 hypomorph resulted in a substantial increase in olaparib resistance (IC50 = 1047 nM), only surpassed by expression of FL BRCA1 (IC50 = 3962 nM) (Figure 2B). Point mutations in the RING (C64R) or BRCT (R1699Q) domains, or a shorter deletion in the RING domain (M48 START), which recapitulate the same protein-binding defects of the more extreme deletions (Figure S1A), as well as the coiled-coil mutation L1407P, also resulted in no to mild increase in resistance to olaparib in our system (Figure 2C). Similar results were obtained in response to carboplatin treatment, where only the expression of FL BRCA1 or the ∆exon11 hypomorph generated resistance. Importantly, and in contrast to the olaparib responses, IC50 values for carboplatin between BTR cells complemented with FL BRCA1 or the ∆exon11 hypomorph were very similar (Figure 2D). Of note, expression of FL or different hypomorphic variants of BRCA1 did not affect proliferation or DNA replication rates (Figure S2G).

Given that the BRCA1 ∆exon11 hypomorph is expressed at significantly higher levels than the FL BRCA1 protein in our system (Figure S2F), we performed a doxycycline dose-titration experiment in these cell lines. While expression of both FL and ∆exon11 BRCA1 reached saturation conditions at around 100 ng/mL doxycycline (Figure 2E), maximal resistance to olaparib was already achieved between 5 and 10 ng/mL doxycycline in the case of FL BRCA1, and between 100 and 500 ng/mL doxycycline in the case of the BRCA1 ∆exon11 hypomorph (Figure 2F). Collectively, these results show that, among all BRCA1 hypomorphs tested, only expression of the ∆exon11 protein results in resistance to olaparib and carboplatin to levels associated with restored HRR. Importantly, however, this is only partially achieved at expression levels significantly higher than those of the FL BRCA1 protein, confirming the hypomorphic nature of the ∆exon11 deletion.

### 3.4. Expression of FL BRCA1 or the BRCA1 ∆exon11 Hypomorph Protein Generates Resistance to Olaparib In Vivo

**Figure 3:**
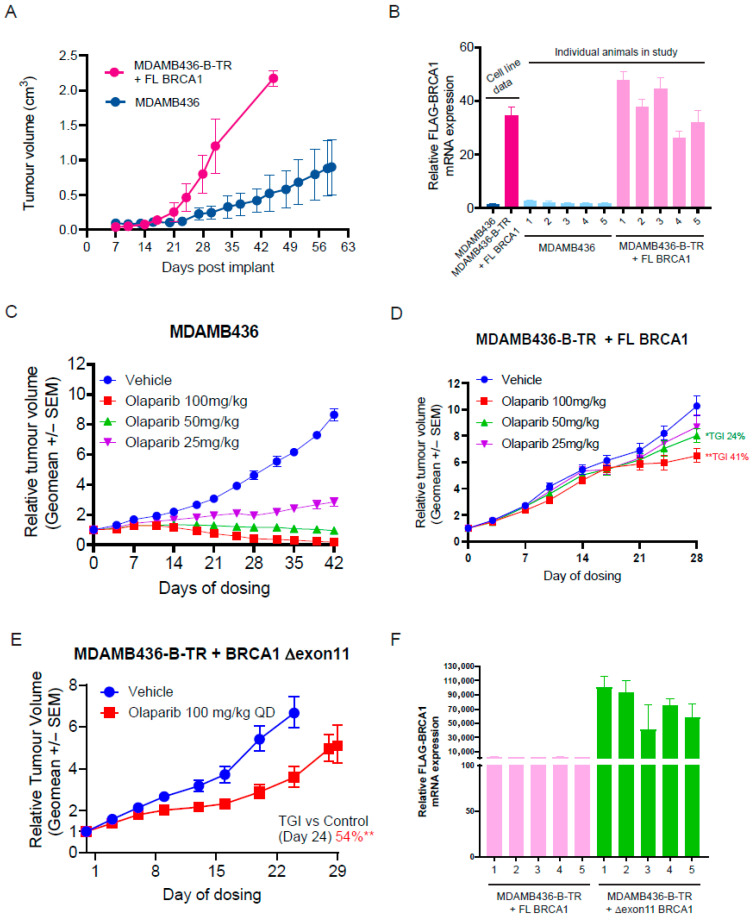
(**A**) Tumour volume growth curves of MDAMB436 (BRCA1 mutant) cells or MDAMB436-B-TR cells expressing full-length (+FL) BRCA1 in SCID mice (mice *n* = 5/group). (**B**) Relative *FLAG-BRCA1* mRNA expression in tumour samples from individual mice from the experiment in A. Expression is compared to samples taken from in vitro culture of the same cell lines and is normalised to that of *GAPDH*. (**C**) Dose–response efficacy of olaparib in the MDAMB436 (BRCA1m) xenograft model (mice *n* = 10/group). (**D**) Dose–response efficacy of olaparib in the MDAMB436-B-TR + full-length (FL) BRCA1 expressing xenograft model (mice *n* = 8/group). (**E**) Dose–response efficacy of olaparib in the MDAMB436-B-TR + ∆exon11 BRCA1 expressing xenograft model (mice *n* = 8/group). Graphs depict the geometrical mean (geomean) of tumour volume ± SEM and percentage TGI (tumour growth inhibition). Statistical significance was evaluated and compared to the vehicle group using a one-tailed *t*-test (mice *n* = 8/group). Statistical significance is indicated as follows: *, *p* ≤ 0.05; **, *p* ≤ 0.01. (**F**) Relative *FLAG-BRCA1* mRNA expression in tumour samples from individual mice implanted with MDAMB436-BTR cells expressing the full-length (FL) or ∆exon11 *BRCA1* constructs. Expression is normalised to that of *GAPDH*. Error bars in tumour measures are ± standard error of the mean (SEM).

We decided to assess whether the increased resistance to olaparib in the BTR complementation system observed in vitro could be translated in vivo. Interestingly, BTR cells complemented with FL BRCA1, implanted as mouse xenografts, developed tumours at a faster rate than parental, BRCA1 mutant MDAMB436 cells (Figure 3A). Analysis of mRNA levels of FLAG-tagged *BRCA1* confirmed its expression in the BTR-derived tumours (Figure 3B). As described before [23], tumours generated after implantation of the parental MDAMB436 cell line showed exquisite sensitivity to olaparib in a dose-dependent manner, with the top dose of olaparib used (the clinically relevant 100 mg/kg dose) causing complete tumour regressions (Figure 3C). Importantly, xenografts of the BTR cell line complemented with FL BRCA1 displayed strong resistance to olaparib, in agreement with our in vitro data, with the 100 mg/kg dose showing a degree of tumour growth inhibition (41% TGI) compared to vehicle control (Figure 3D). Also correlating with the in vitro data, xenografts of the BTR cell line expressing the BRCA1 ∆exon11 hypomorph showed increased resistance to olaparib, but to a lesser extent than their FL BRCA1 complemented counterparts (54% TGI at 100 mg/kg; Figure 3E). Interestingly, expression of the FLAG-tagged ∆exon11 *BRCA1* transcript was significantly higher than that of the FL transcript (Figure 3F), suggesting that increased expression of the BRCA1 ∆exon11 protein could be due to increased mRNA levels, as reported before [14].

Taken together, these data show that the BTR complementation approach is a useful in vivo system to model acquired resistance to olaparib caused by expression of BRCA1 protein variants.

### 3.5. Combination of Olaparib and the ATR Inhibitor, Ceralasertib, Increases Efficacy in Cell Lines Expressing the BRCA1 ∆exon11 Hypomorph

**Figure 4:**
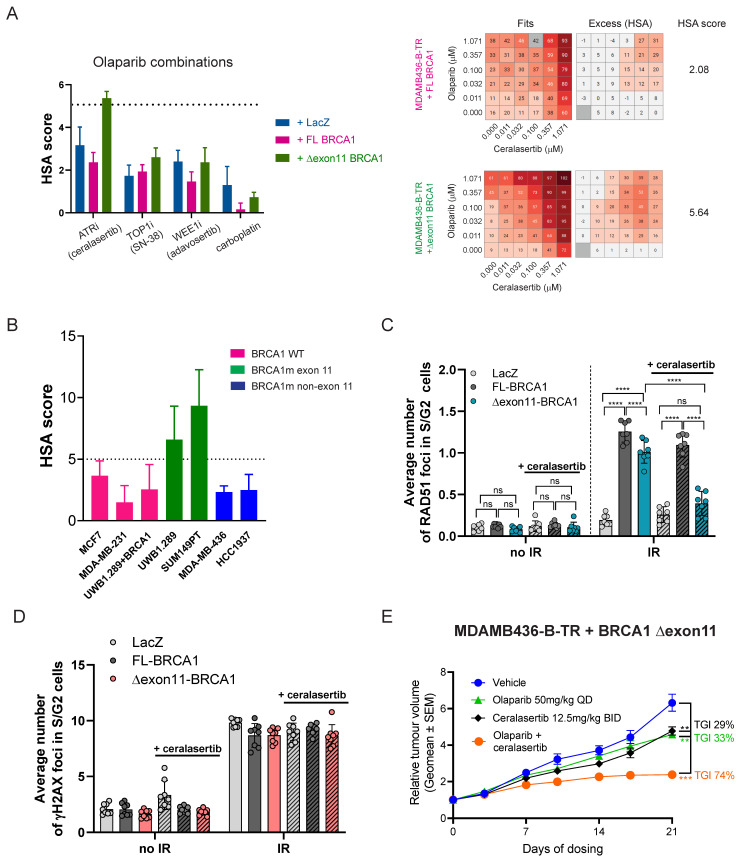
(**A**). (**Left panel**): combination activity between olaparib and ceralasertib, SN-38, adavosertib, or carboplatin in MDAMB436-B-TR cells expressing full-length (+FL) ∆exon11 or no BRCA1 (+LacZ) protein. Scores above 5 (marked by the dotted line on the graph) are described as synergistic based on the highest single agent (HSA) model. (**Right panel**): representative examples of 6 × 6 combination matrices between olaparib and ceralasertib in MDAMB436-B-TR cells expressing full-length (+FL) BRCA1 (top) or ∆exon11 BRCA1 (bottom). In the Fits panels, values between 0 and 100 represent cytostatic effects. The excess (HSA) panels represent the values used to calculate synergy scores. Values coloured in pink/brown represent positive interactions. (**B**) combination activity between olaparib and ceralasertib in cell lines carrying a WT copy of *BRCA1* (MCF7, MDAMB231, UWB1.289 + BRCA1), a *BRCA1* gene with a pathogenic mutation inside the exon 11 of the gene (UWB1.289, SUM149PT) or a *BRCA1* gene with a pathogenic mutation outside of the exon 11 of the gene (MDAMB436, HCC1937). Scores above 5 (marked by the dotted line on the graph) are described as synergistic based on the HSA model. (**C**) Quantification of RAD51 foci formation in MDAMB436-B-TR cells expressing full-length (+FL) ∆exon11 or no BRCA1 (+LacZ) protein, irradiated (IR) or not with 5 Gy, and treated or not with ceralasertib (0.3 µM for 24 h prior to IR). Statistical analysis was performed using One-Way ANOVA with Sidak multiple comparisons, *n* = 3 biological replicates with 2–3 technical replicates in each experiment: ns—not significant, **** *p* < 0.0001. (**D**) Quantification of γH2AX foci formation in MDAMB436-B-TR cells expressing full-length (+FL) ∆exon11 or no BRCA1 (+LacZ) protein, irradiated (IR) or not with 5 Gy, and treated or not with ceralasertib (0.3 µM for 24 h). Statistical analysis was performed using One-Way ANOVA with Sidak multiple comparisons, *n* = 3 biological replicates with 2–3 technical replicates in each experiment. (**E**) Efficacy of olaparib, ceralasertib, or their combination in the MDAMB436-B-TR + ∆exon11 BRCA1 expressing xenograft model. The graph depicts the geometrical mean (geomean) of tumour volume ± SEM and percentage TGI (tumour growth inhibition). Statistical significance was evaluated compared to the vehicle group using a one-tailed *t*-test (mice *n* = 10/group). Statistical significance is indicated as follows: **, *p* ≤ 0.01; ***, *p* ≤ 0.001.

Combinations of PARPi with other DDR-targeted agents, as well as with DNA-damaging chemotherapies, are being pursued as a way to overcome PARPi resistance in the clinic [26]. Having established an in vivo system to understand BRCA1 hypomorph-mediated PARPi resistance, we next explored therapeutic approaches to overcome it. With that goal, we assessed the combination activity of olaparib with the ATR inhibitor, ceralasertib [27], the WEE1 inhibitor, adavosertib [28], the DNA crosslinking agent, carboplatin, and the DNA topoisomerase I inhibitor, SN-38, in BTR cells expressing FL BRCA1 or the BRCA1 ∆exon11 hypomorph. As controls, we compared the activity of such combinations in non-complemented BTR cells (LacZ). While the combination activity between olaparib and adavosertib, carboplatin, or SN-38 was very similar between LacZ, FL, or ∆exon11 expressing BTR cell lines, as assessed by the highest single agent (HSA) model [29], the ceralasertib combination provided higher score values in BRCA1 ∆exon11-expressing cells (Figure 4A).

To extend this observation beyond our BTR complementation system, we tested combination of olaparib and ceralasertib in cancer cell lines carrying a wild-type version of *BRCA1* (MCF7, MDAMB231, UWB1.289 + BRCA1), or mutations in *BRCA1* outside (MDAMB436, HCC1937) or inside the exon 11 of the gene (UWB1.289, SUM149PT), with the latter known to express the BRCA1 ∆exon11 hypomorph [22,30]. Consistently, higher HSA scores were observed in the UWB1.289 and SUM149PT cell lines expressing the BRCA1 ∆exon11 hypomorph (Figure 4B). Importantly, expression of the WT version of BRCA1 in the ∆exon11 hypomorph-expressing UWB1.289 cell line reduced efficacy of the combination, in agreement with the data in our BTR complementation system (Figure 4B).

Resistance to PARPi in BRCA1 mutant cell lines and tumours caused by acquired expression of BRCA1 hypomorphic or FL proteins is associated with restored HRR, as measured by the ability of cells to form RAD51 foci in response to DNA damage [1,12]. We compared the ability of non-complemented (LacZ), FL, and ∆exon11 BRCA1 BTR cells to form RAD51 foci in response to ionising radiation (IR) and observed that while LacZ cells failed to show recruitment of RAD51 to DNA damage sites, both FL and ∆exon11 BRCA1 BTR cells effectively formed RAD51 foci (Figure 4C). Interestingly, ∆exon11 BRCA1 BTR cells showed a small but significant defect in RAD51 foci formation when compared to FL BRCA1 complemented cells (Figure 4C), indicative of suboptimal HRR proficiency and reflective of the less pronounced resistance of ∆exon11 BRCA1 BTR cells to olaparib when compared to FL BRCA1 complemented cells, both in vitro and in vivo (Figure 2B and Figure 3D,E). Strikingly, treatment with the ATRi, ceralasertib, strongly reduced the ability of ∆exon11 BRCA1 BTR cells to form RAD51 foci in response to IR, while the effect was much more modest in cells complemented with FL BRCA1 (Figure 4C). Reduced levels of RAD51 foci were not due to decreased DNA damage formation, as assessed by comparable levels of the DNA damage-induced Ser-139 phosphorylation of histone variant H2A.X (γH2AX) in all cell lines exposed to IR in the presence or absence of ceralasertib treatment (Figure 4D). Importantly, combination activity between olaparib and ceralasertib was also observed in vivo, with a significant increase in TGI in ∆exon11 BRCA1 BTR cell xenografts compared to either monotherapy (Figure 4E).

Collectively, these data show increased activity in vitro and in vivo of the combination of olaparib and ceralasertib in cell lines expressing the BRCA1 ∆exon11 hypomorph, which can be explained by the requirement of ATR activity for RAD51 foci formation in response to DNA damage in the presence of the BRCA1 ∆exon11 hypomorph.

## 4. Conclusions

Although there are literature examples linking the expression of BRCA1 hypomorphs with resistance to chemotherapy and PARPi, both in vitro and in vivo, our work provides the most detailed analyses of BRCA1 hypomorphic variants to date. Through the generation of an allelic series, including clinically relevant point mutations and deletions, we uncover that the hypomorphic nature of the described ∆exon11 variants may reside in their inability to recapitulate the full extent of BRCA1 protein–protein interactions, even under overexpression conditions. Importantly, we also describe a clear dose–response behaviour between BRCA1 ∆exon11 protein expression levels and response to PARPi, which may help explain the apparent lack of correlation between the ability of BRCA1 mutant PDX models expressing BRCA1 ∆exon11 hypomorphs to form BRCA1 and RAD51 foci, and their response to PARPi [15,31]. In addition, our analyses point towards the actual length of the deletion inside the exon 11 of *BRCA1*, rather than any specific regions encoded within, as the main determinant of the hypomorphic nature of these variants, describing a new potential function for this predominantly unstructured domain [32]. This could explain why, despite the *BRCA1* exon 11 region being the most flexible at accommodating large deletions as secondary reversion mutations potentially driving resistance to chemotherapy and PARPi in patients, deletions resulting in loss of more than 300 amino acids inside the exon 11 sequence are extremely rare events [33]. Our efforts to produce a doxycycline-inducible BRCA1 complementation system, usable both in vitro and in vivo, have been key to generating these novel insights.

Our analyses also demonstrate that mutations or deletions blocking the ability of BRCA1 to interact with its partners through the RING, coiled coil, or BRCT domains without overtly impacting protein stability all have a drastic effect on BRCA1 function at preventing heightened sensitivity to carboplatin and olaparib. In that regard, only overexpression of the ∆exon11 version of BRCA1 generated resistance to olaparib with an IC50 value (approximately 1 μM) above the minimal free concentration of olaparib (approximately 300 nM) in plasma of patients on the established monotherapy dose of 300 mg twice daily [34], and generated levels of resistance to carboplatin very similar to those produced by expression of the full length protein. The apparent different behaviour of the BRCA1 ∆exon11 hypomorph versus the full-length protein between the olaparib (partial resistance) and carboplatin (full resistance) experiments may be driven by the reduced therapeutic index of carboplatin between fully HRR-proficient and partially proficient settings.

Expression of RING and BRCT mutant versions of BRCA1 has been linked to chemotherapy and PARPi resistance in genetically engineered mouse models of BRCA1 deficiency [10] and in PDX models [12,17]. Our data are in accordance with previous reports showing that overexpression of RING or BRCT mutant BRCA1 proteins caused modest resistance to chemotherapy and PARPi, both in vitro and in vivo [11,35], even at expression levels higher than those of the FL protein (Figure S2F). Given the clear hypomorphic character of these mutants, we hypothesize that additional mutations and/or DNA repair pathway adaptations are required in tumours for these RING and BRCT hypomorphs to drive clinically meaningful therapy resistance [12,35], making this an area where further research is required.

Our data also highlight that the hypomorphic nature of BRCA1 ∆exon11 can be explained, at least in part, by its inability, even at high overexpression levels, to fully complement the deficiency in RAD51 foci formation caused by BRCA1 loss. This can be linked to our observation of an impaired capacity of BRCA1 ∆exon11 to interact with PALB2, an interaction that is essential to bring the PALB2-BRCA2-RAD51 complex to sites of DNA damage [7]. Although a connection between the expression of BRCA1 ∆exon11 and resistance to PARPi has been described before [14,35], there has been no direct correlation established between the presence of the hypomorph and resistance in different studies using PDX models [12,15]. Our data strongly suggest that BRCA1 ∆exon11 protein expression levels are the key determinant of PARPi-resistance in these circumstances.

Importantly, we uncover a specific reliance of our MDAMB436-BTR BRCA1 ∆exon11 system on the activity of the DNA damage checkpoint kinase ATR to effectively recruit RAD51 to DNA damage foci. The reason why the BRCA1 ∆exon11 hypomorph is specifically reliant on ATR activity to exert its HRR function is unclear, but it is important to note that there is a cluster of a dozen ATM/ATR phosphorylation sites on BRCA1 in the PALB2 interacting region located outside the exon 11 of the gene [36]. Regardless of the reason, this vulnerability could potentially explain the increased combination activity of ceralasertib plus olaparib in cell lines expressing the BRCA1 ∆exon11 hypomorph in vitro, which translated into significantly improved efficacy for the combination over either monotherapy in vivo, as has been observed in PDX models expressing the BRCA1 ∆exon11 hypomorph [3]. As the combination of ceralasertib and olaparib is being tested in the clinic [37], supported by pre-clinical data showing activity of this combination in HRR-deficient settings and beyond [2,3], our work provides a supportive mechanistic platform to help explain the benefit of this combination in patients with tumours harbouring mutations in the exon 11 of *BRCA1*.

## Figures and Tables

**Table 1:** Antibodies used for western blotting.

Target	Host Species	Catalogue Number	Provider	Working Dilution (WB)
Anti-Mouse IgG HRP	Goat	7076	Cell Signalling Tech (Danvers, MA, USA)	1:2000–1:3000
Anti-Rabbit IgG HRP	Goat	7074	Cell Signalling Tech	1:2000–1:3000
BARD1	Rabbit	ab226854	Abcam (Cambridge, UK)	1:1000
BRCA1	Mouse	OP92	Merck (Rahway, NJ, USA)	1:500–1:1000
BRCA1	Rabbit	07-434	Merck	1:500–1:1000
BRIP1	Rabbit	4578	Cell Signalling Tech	1:1000
CTIP (RBBP8)	Rabbit	9201	Cell Signalling Tech	1:1000
GAPDH	Rabbit	2118	Cell Signalling Tech	1:1000
PALB2	Rabbit	A301-246A	Bethyl Laboratories (Montgomery, TX, USA)	1:1000
RAD51B	Rabbit	GTX40242	Genetex (Irvine, CA, USA)	1:1000
Tet Repressor	Mouse	TET01	MoBiTec (Eupen, Belgium)	1:1000
Vinculin	Mouse	V9131	Sigma-Aldrich (St. Louis, MO, USA)	1:2000–1:5000

**Table 2:** Oligonucleotides used for RT-qPCR.

Target Gene	Orientation	Sequence (5′-3′)
*BRCA1*	Forward	TGGGTGTTGGACAGTGTAGC
	Reverse	TCGTCGTCATCCTTGTAATCGT
*GAPDH*	Forward	ATGACATCAAGAAGGTGGC
	Reverse	CATACCAGGAAATGAGCTTG

**Table 3:** Antibodies used for immunofluorescence experiments.

Target	Host Species	Catalogue Number	Provider	Working Dilution (IF)
Alexa Fluor 488	Goat (anti-mouse)	A-11029	Thermo Fisher Scientific	1:2000
Alexa Fluor 594	Goat (anti-rabbit)	A-11037	Thermo Fisher Scientific	1:2000
γH2AX	Mouse	05-636	Millipore (Sigma/Merck)	1:5000
RAD51	Rabbit	70-001	Bioacademia	1:7000

## Data Availability

The original contributions presented in this study are included in the article/Supplementary Materials. Further inquiries can be directed to the corresponding author.

## Supplementary Materials

The following supporting information can be downloaded at: https://www.mdpi.com/article/10.3390/cancers18020309/s1, Figure S1: (A) Co-immunoprecipitation experiments in HEK293T cells with the different forms of BRCA1 used in this study. UT: unstransfected; FL: full length; I: input; A: pull down with protein A beads; F: pull down with FLAG beads. (B) Quantification of the relative BRCA1 protein pull down normalized to the BRCA1 full length (FL) protein, related to Figure 1D. (C) Schematic of the BRCA1 exon 11 protein deletions analysed in this study. (D) Co-immunoprecipitation experiments in HEK293T cells with the different exon 11 deletions of BRCA1 used in this study. UT: unstransfected; FL: full length; I: input; F: pull down with FLAG beads. Figure S2: (A) Genetic alterations in the MDAMB436 cell line, as described in the Cancer Cell Line Encyclopaedia data update of 2019. (B) Western blot showing expression of RAD51B only in MDAMB436 cells stably transfected with a RAD51B expression construct. Vinculin was used as loading control. (C) Western blot showing expression of the tetracycline repressor (TetR) only in MDAMB436 or MDAMB436-B (+RAD51B) cells transfected with the TetR construct (+). Vinculin was used as loading control. (D) Western blot showing expression of FLAG-tagged BRCA1 in the presence of tetracycline induction in MDAMB436-TR (+TetR) or MDAMB436-B-TR (+RAD51B +TetR) cells. GAPDH was used as loading control. (E) Dose-response curves of olaparib in colony formation assays in tetracycline-repressor (TR) expressing MDAMB436-B (+RAD51B) cells with (+BRCA1) or without (+LacZ) BRCA1 complementation, cultured in the presence or absence of doxycycline (+/− dox). (F) Western blot showing immunoprecipitation of FLAG-tagged BRCA1 constructs (or LacZ control) stably transfected in MDAMB436-B-TR cells, in the presence or absence of doxycycline induction. FL: full length. GAPDH was used as input control. File S1: The original western blot figures.
