# Supplementary material for: An Inducible BRCA1 Expression System with In Vivo Applicability Uncovers Activity of the Combination of ATR and PARP Inhibitors to Overcome Therapy Resistance
Source: Cancers (Basel). 2026 Jan 20;18(2):309. doi: 10.3390/cancers18020309 (PMC12838977; doi:10.3390/cancers18020309)
Supplement: Supplementary file 1 [file cancers-18-00309-s001.zip › cancers-4069969-supplementary/cancers-4069969-File S1. The original western blot figures.pdf]

Figure 1 – B/C

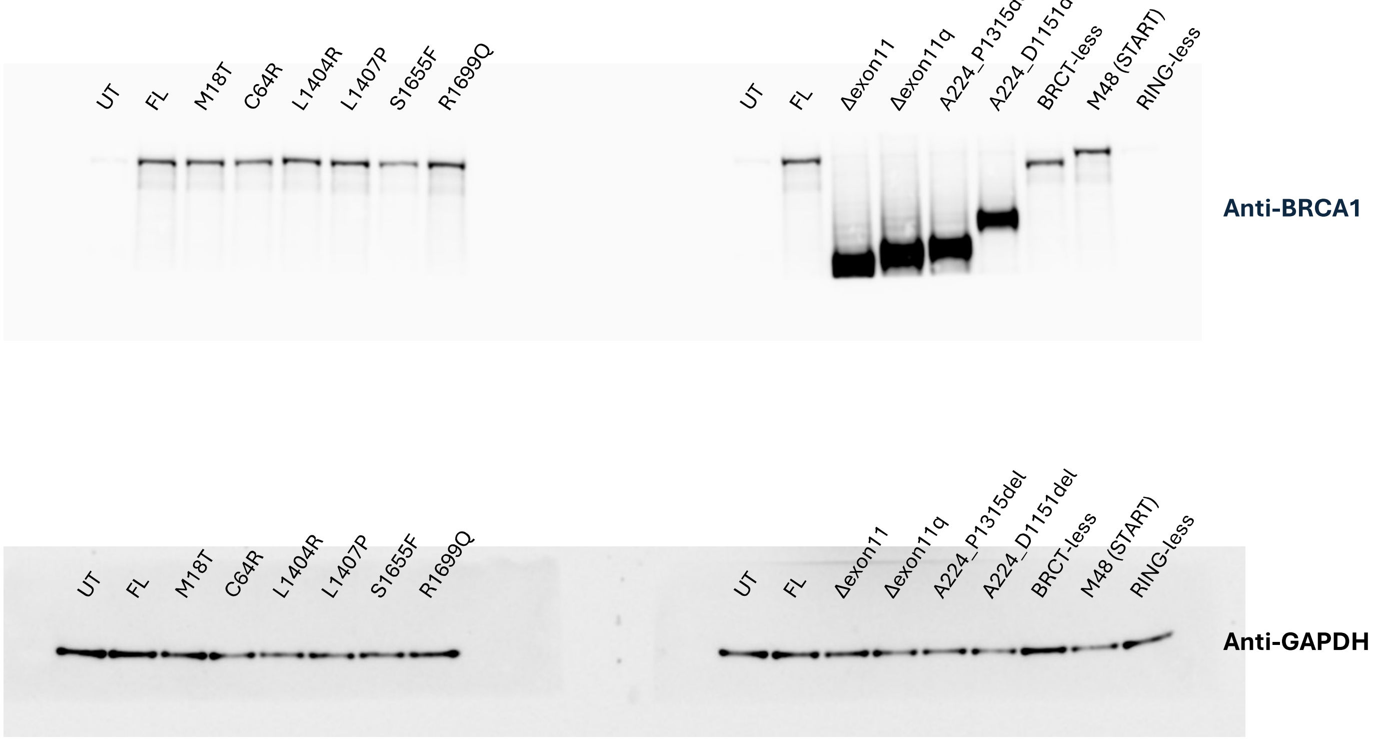
UT
FL
M18T
C64R
L1404R
L1407P
S1655F
R1699Q
UT
FL
Δexon11
Δexon11q
A224_P1315del
A224_D1151del
BRCT-less
M48 (START)
RING-less
Anti-BRCA1
UT
FL
M18T
C64R
L1404R
L1407P
S1655F
R1699Q
UT
FL
Δexon11
Δexon11q
A224_P1315del
A224_D1151del
BRCT-less
M48 (START)
RING-less
Anti-GAPDH

Figure 1 – D (10 second exposure)

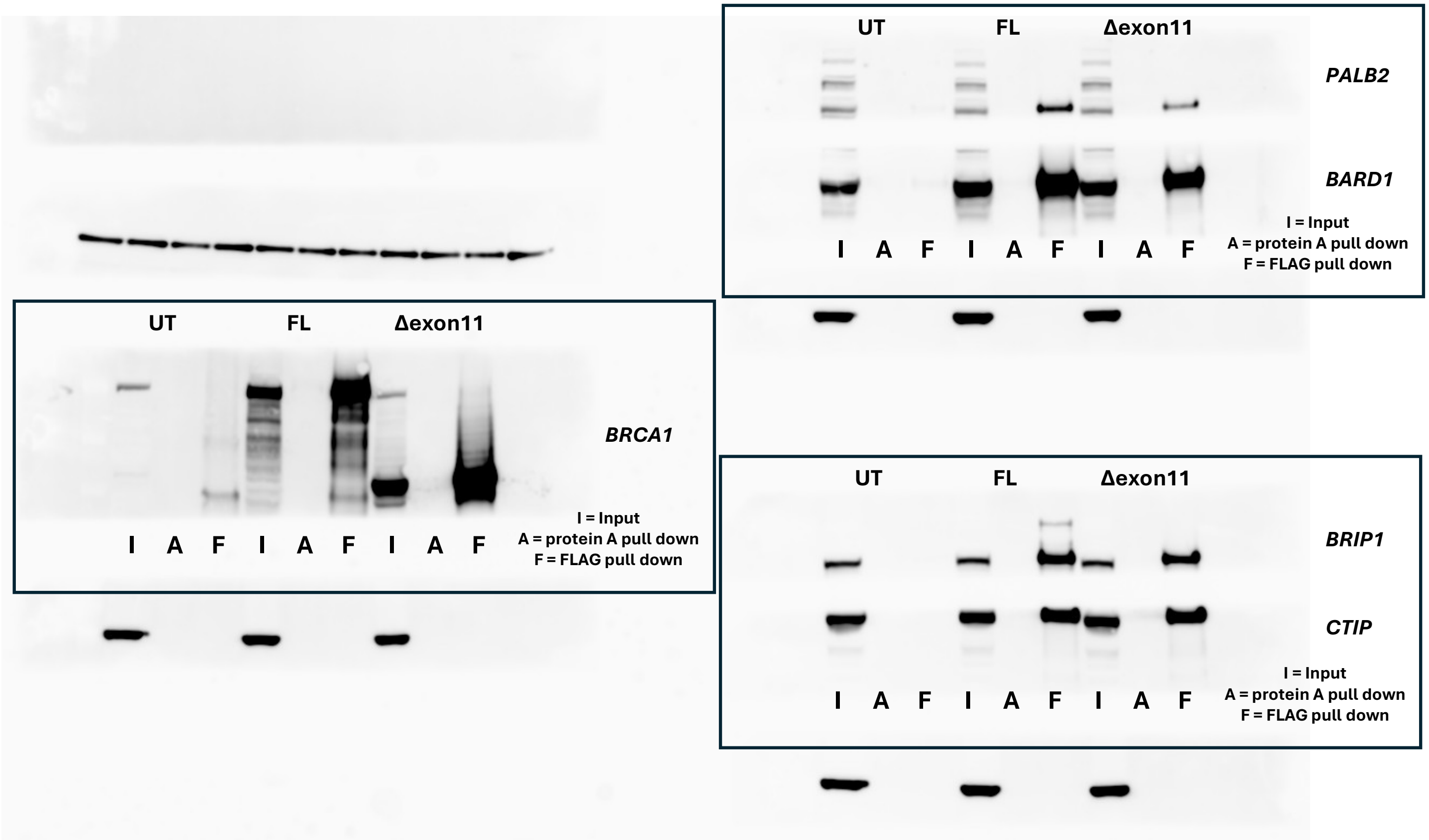

Figure 1 – D (1 minute exposure)

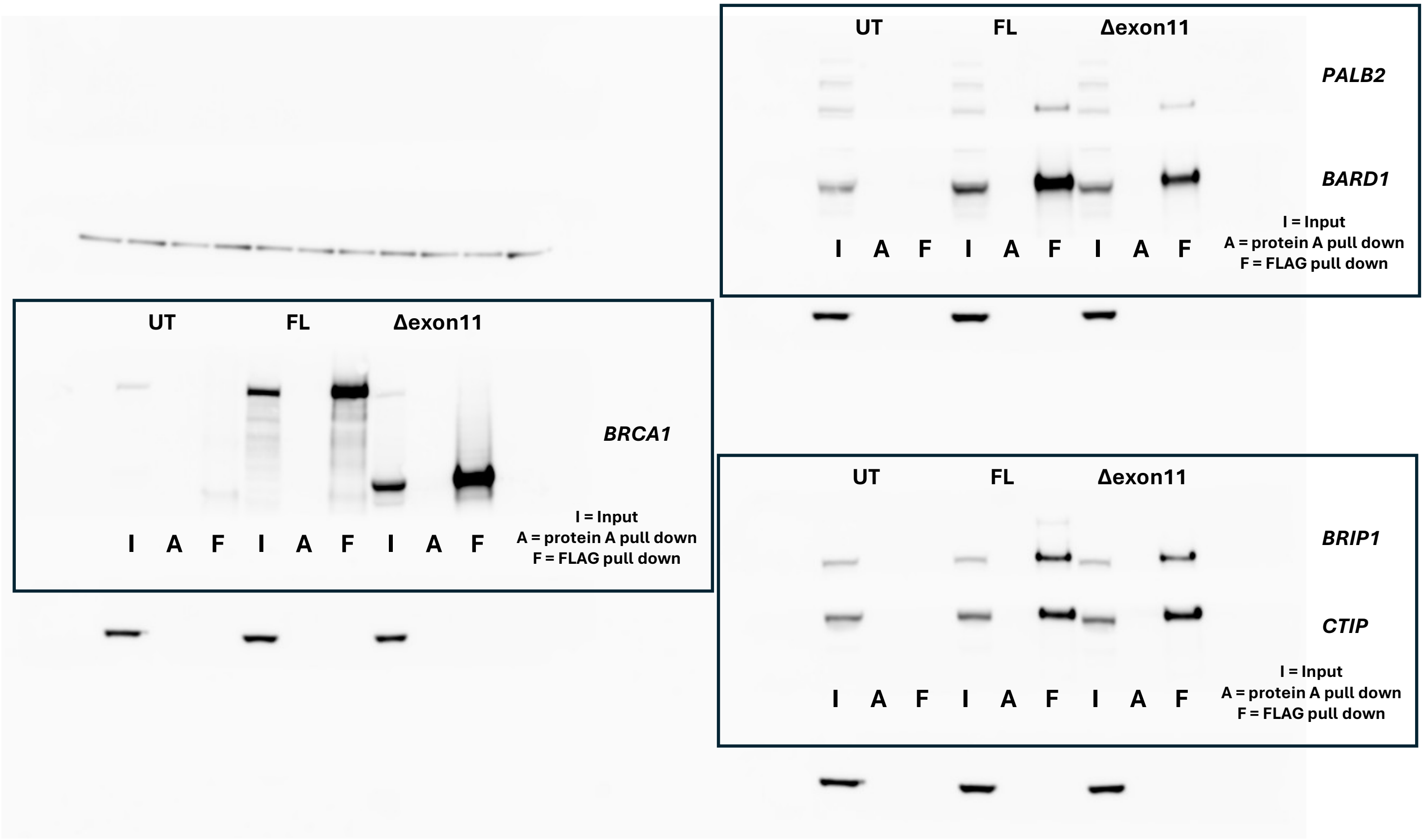

Figure 1 – D (5 minute exposure)

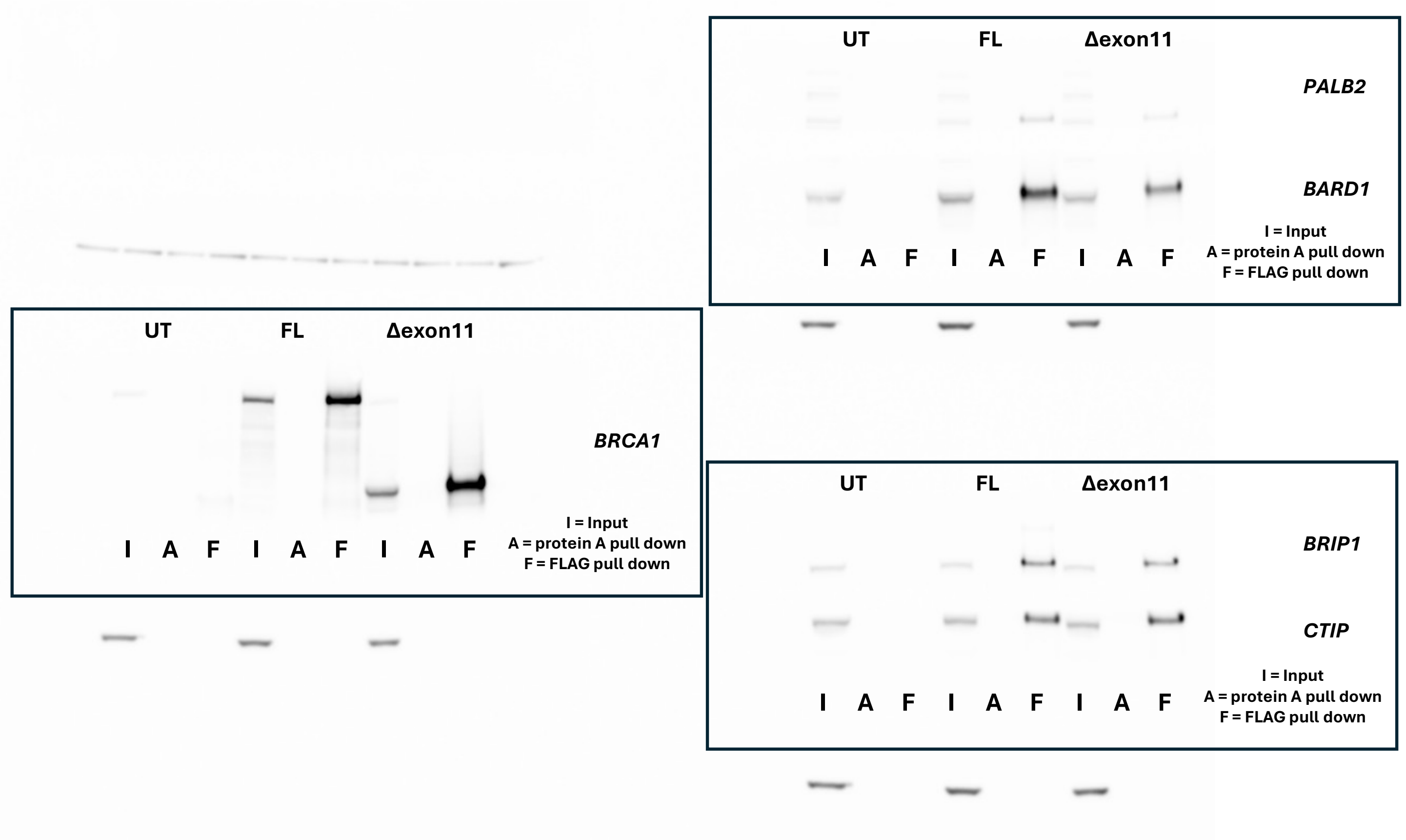

Figure 2 – E

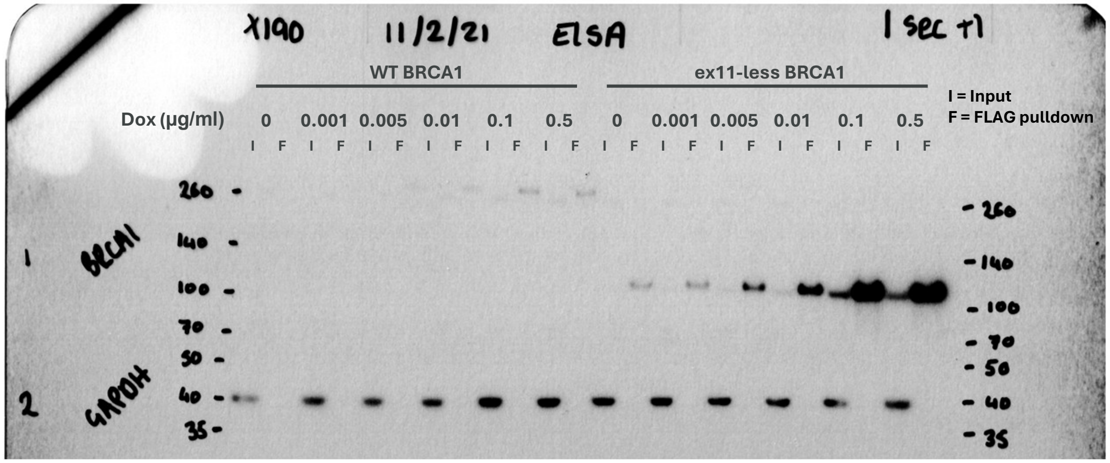
X190 11/2/21 ELSA 1 sec +1
WT BRCA1
ex11-less BRCA1
I = Input
F = FLAG pulldown
Dox (µg/ml)
0 0.001 0.005 0.01 0.1 0.5 0 0.001 0.005 0.01 0.1 0.5
I F I F I F I F I F I F I F I F I F I F I F I F
1 BRCA1
2 GAPDH
260
140
100
70
50
40
35

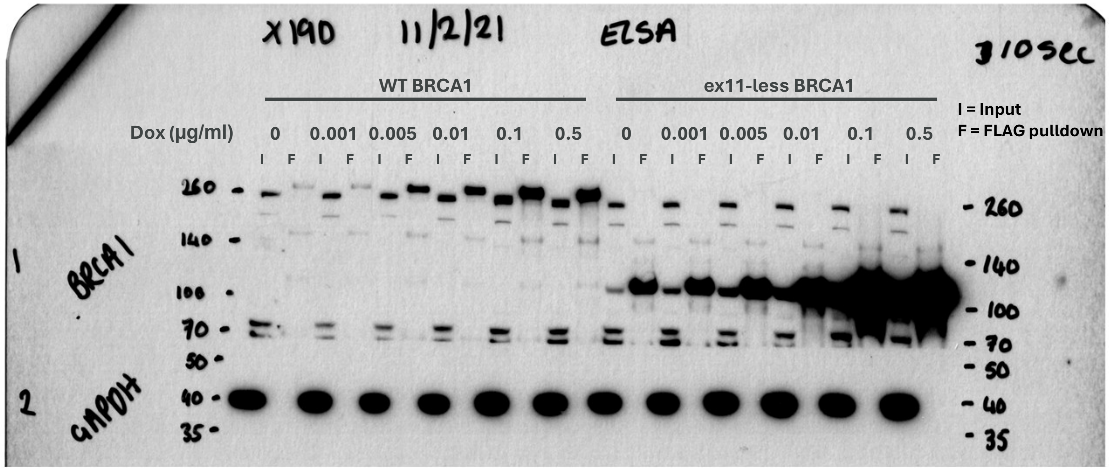
X190 11/2/21 ELSA 10 sec
WT BRCA1
ex11-less BRCA1
I = Input
F = FLAG pulldown
Dox (µg/ml)
0 0.001 0.005 0.01 0.1 0.5 0 0.001 0.005 0.01 0.1 0.5
I F I F I F I F I F I F I F I F I F I F I F I F
1 BRCA1
2 GAPDH
260
140
100
70
50
40
35

Figure S1 – A (various exposures)

I = Input
A = protein A pull down
F = FLAG pull down

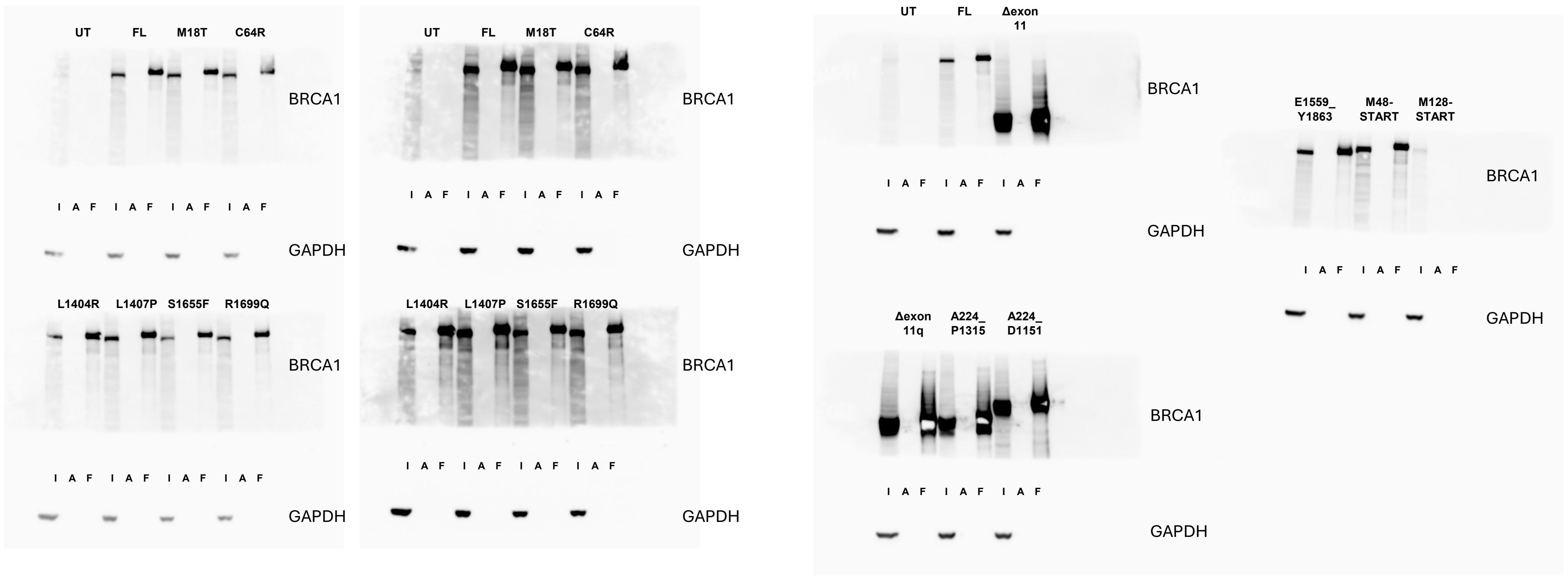

Figure S1 – A (various exposures)

I = Input
A = protein A pull down
F = FLAG pull down

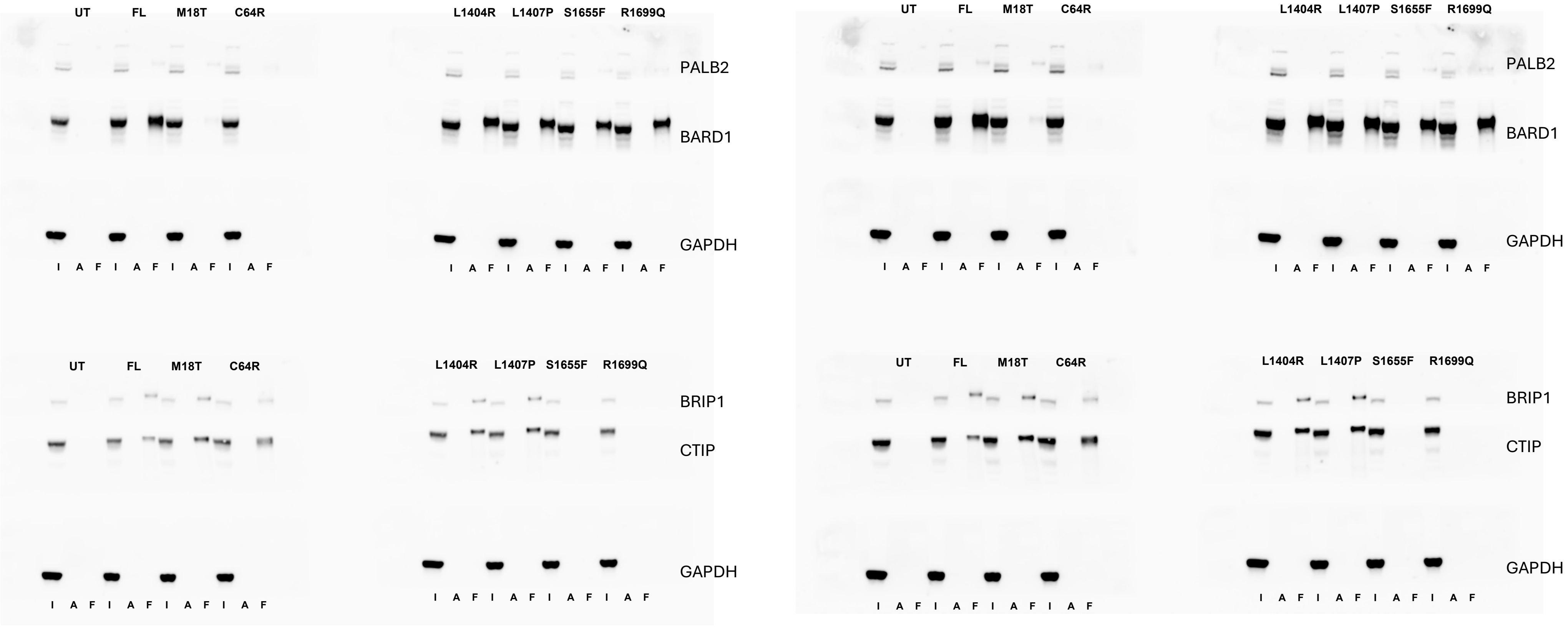

Figure S1 – A (various exposures)

I = Input
A = protein A pull down
F = FLAG pull down

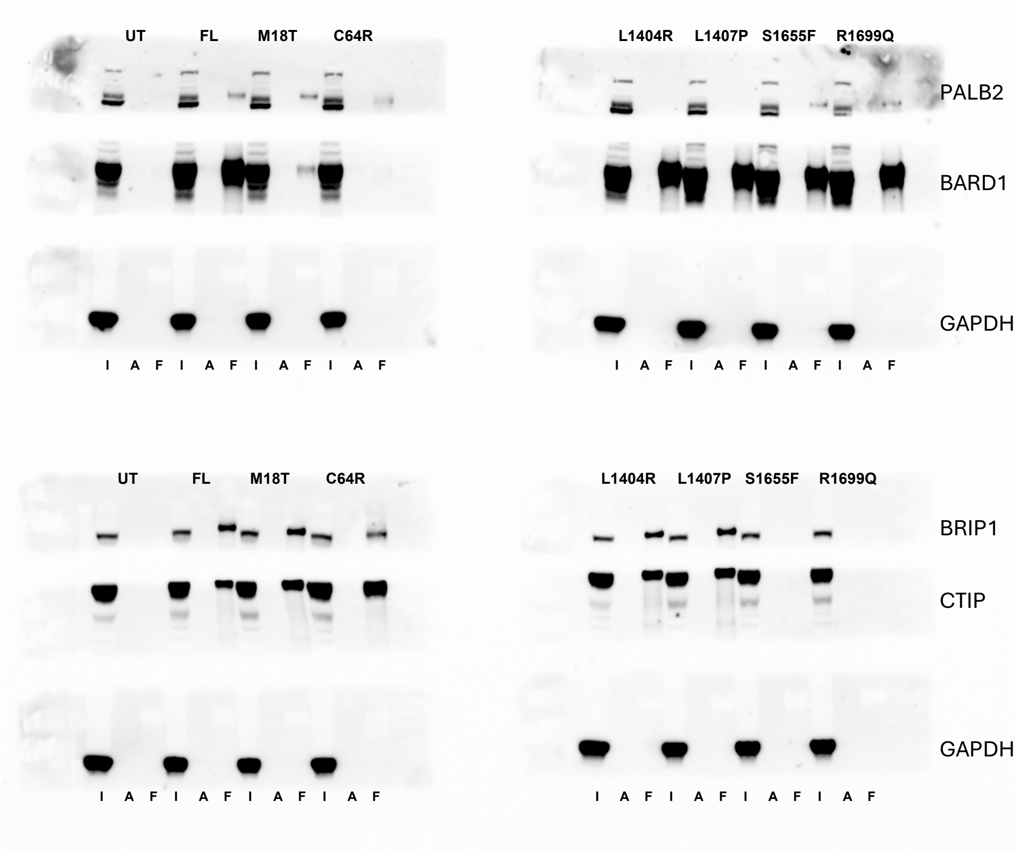

Figure S1 – A (various exposures)

I = Input
A = protein A pull down
F = FLAG pull down

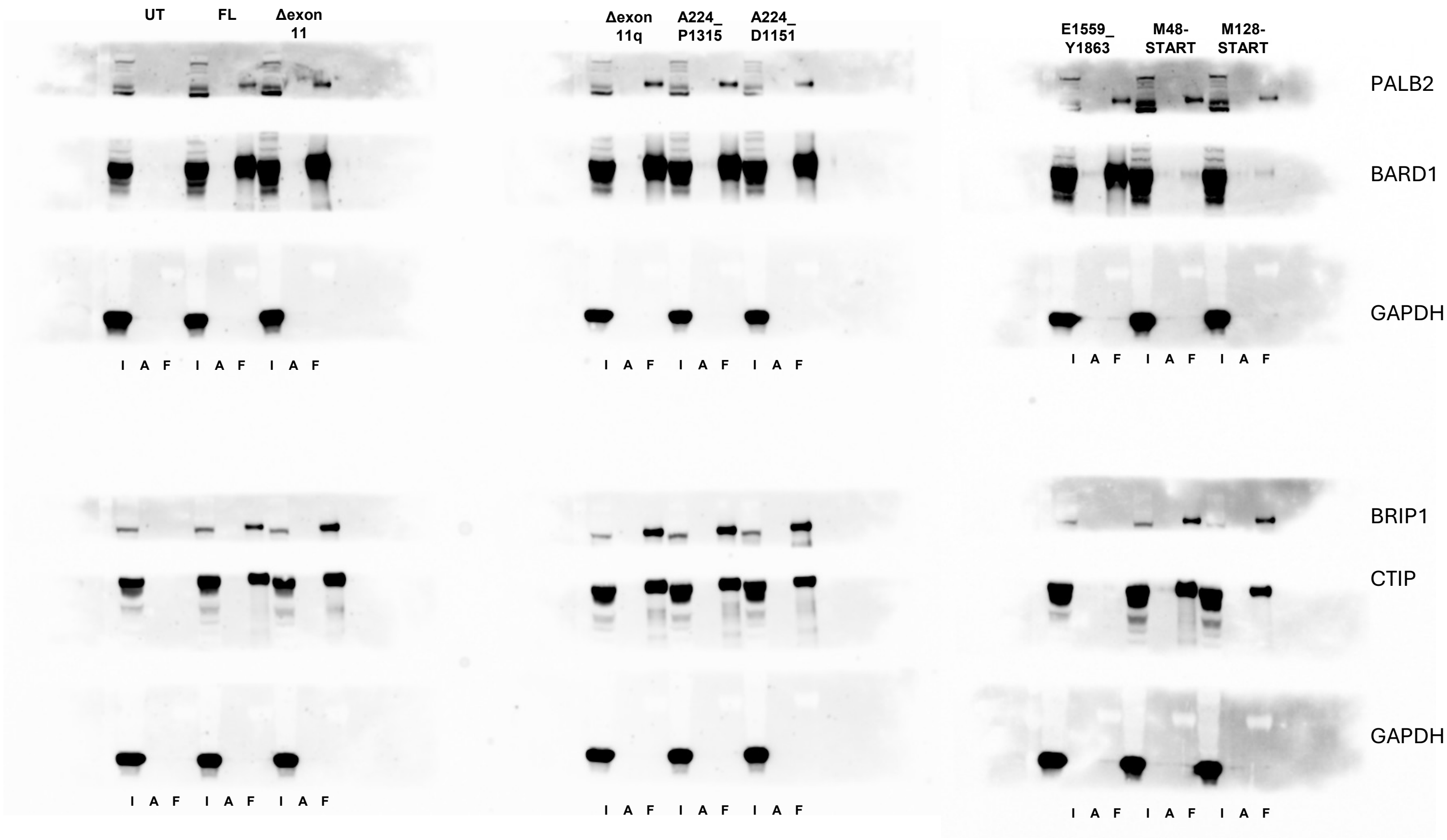

Figure S1 – A (various exposures)

I = Input
A = protein A pull down
F = FLAG pull down

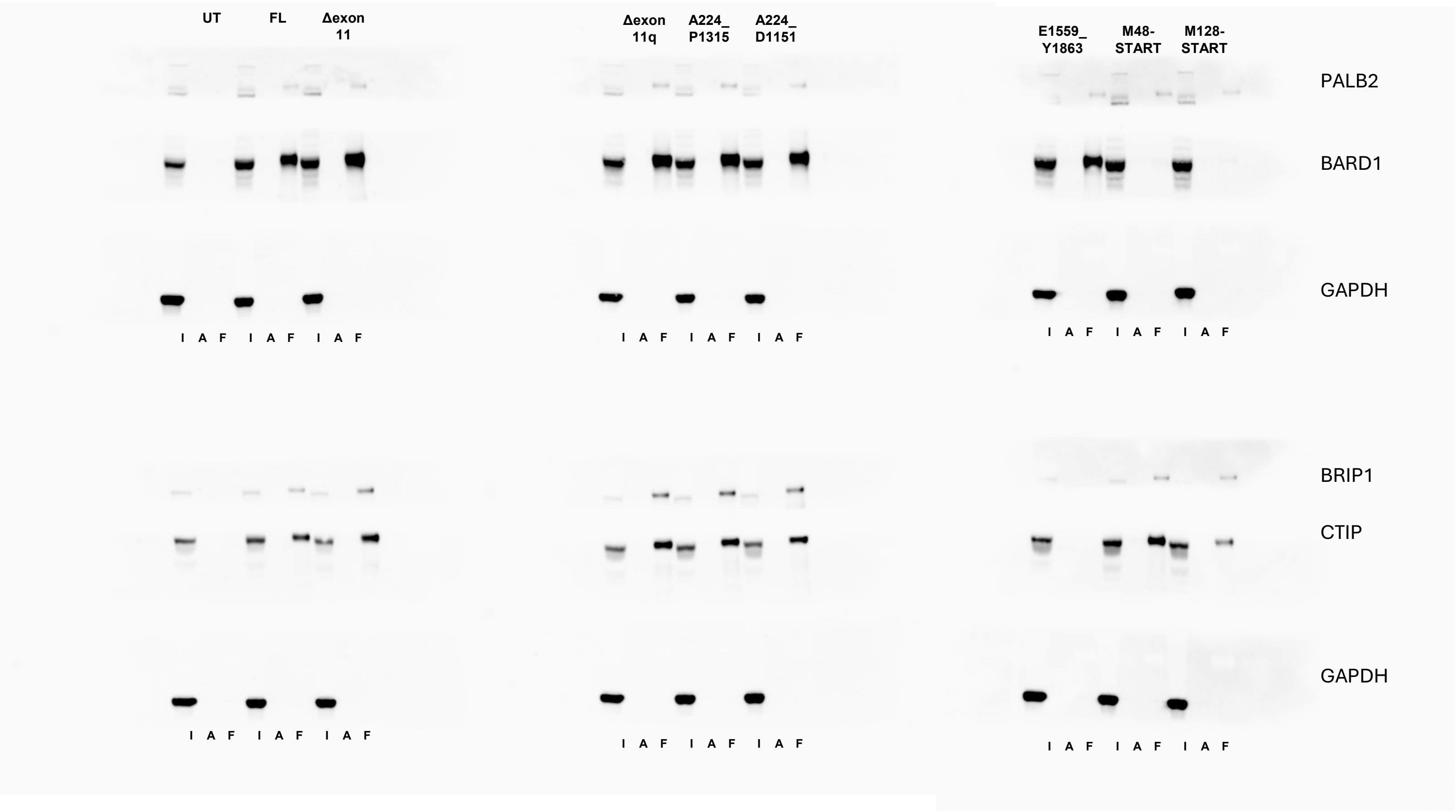

Figure S1 – A (various exposures)

I = Input
A = protein A pull down
F = FLAG pull down

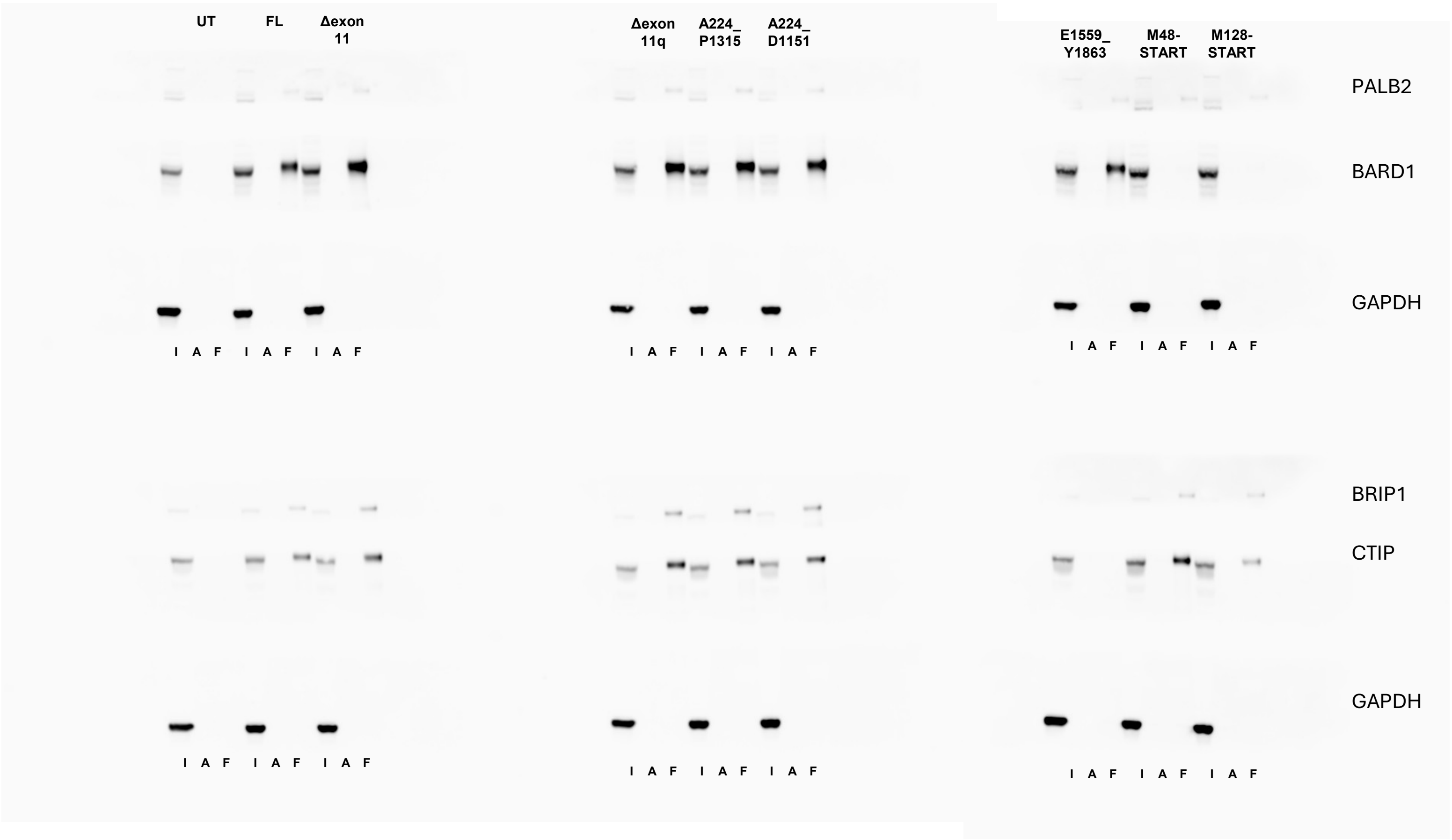

Figure S1 – D (30 second exposure)

I = Input, F = FLAG pull down

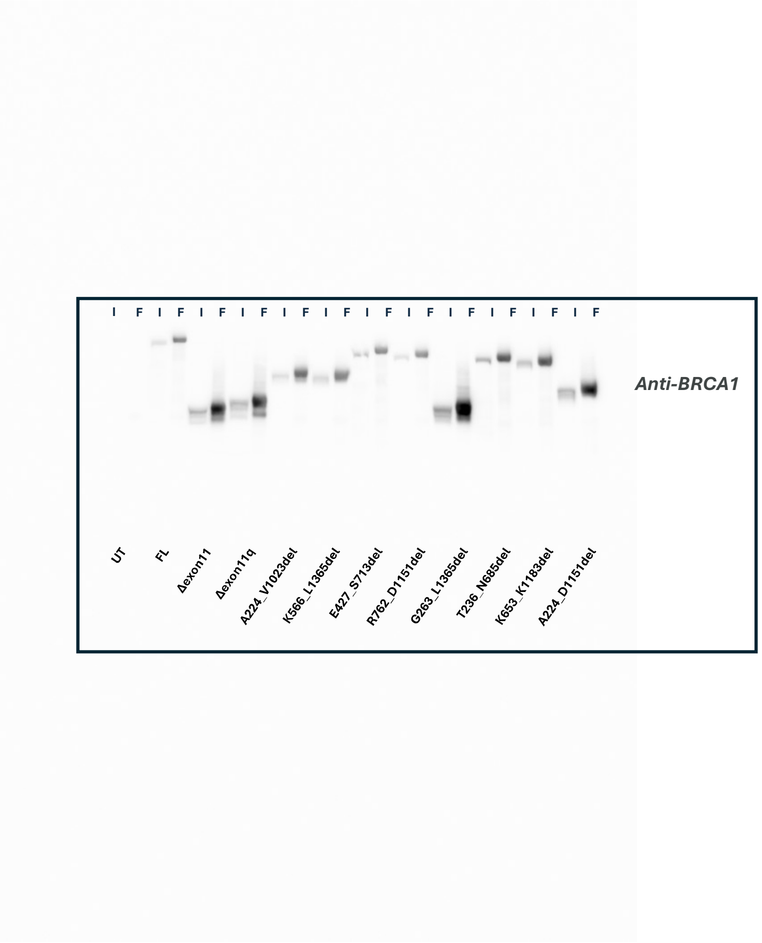

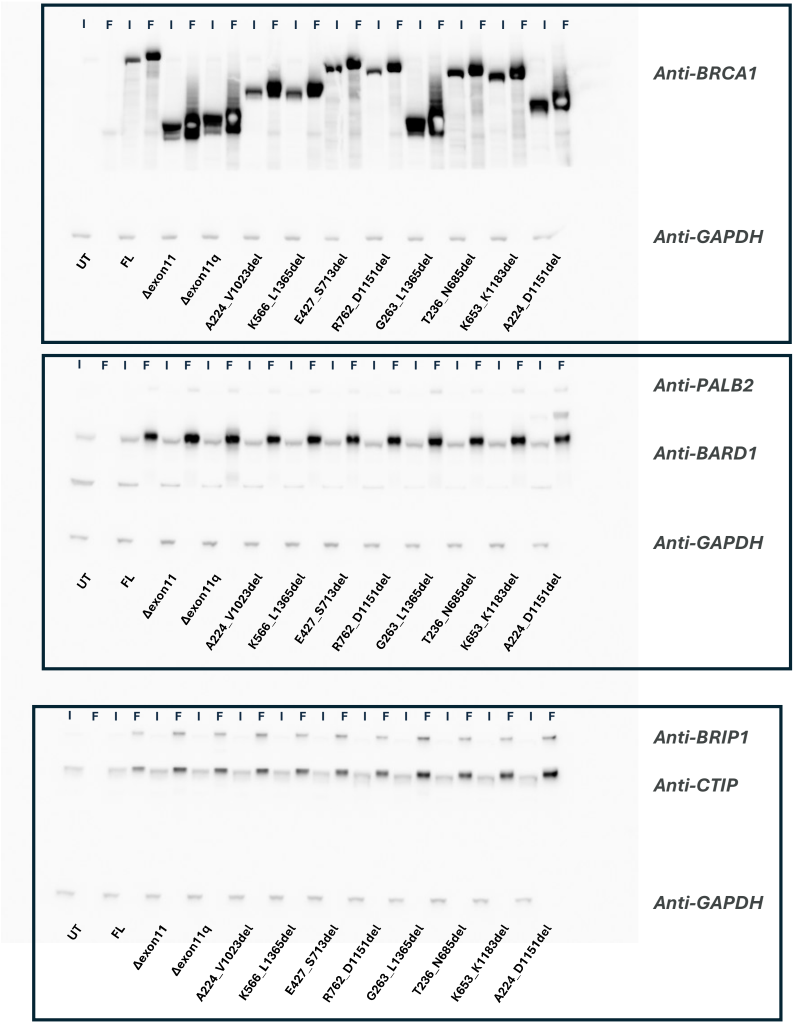

Figure S1 – D (2/5/15 minute exposure)

I = Input, F = FLAG pull down

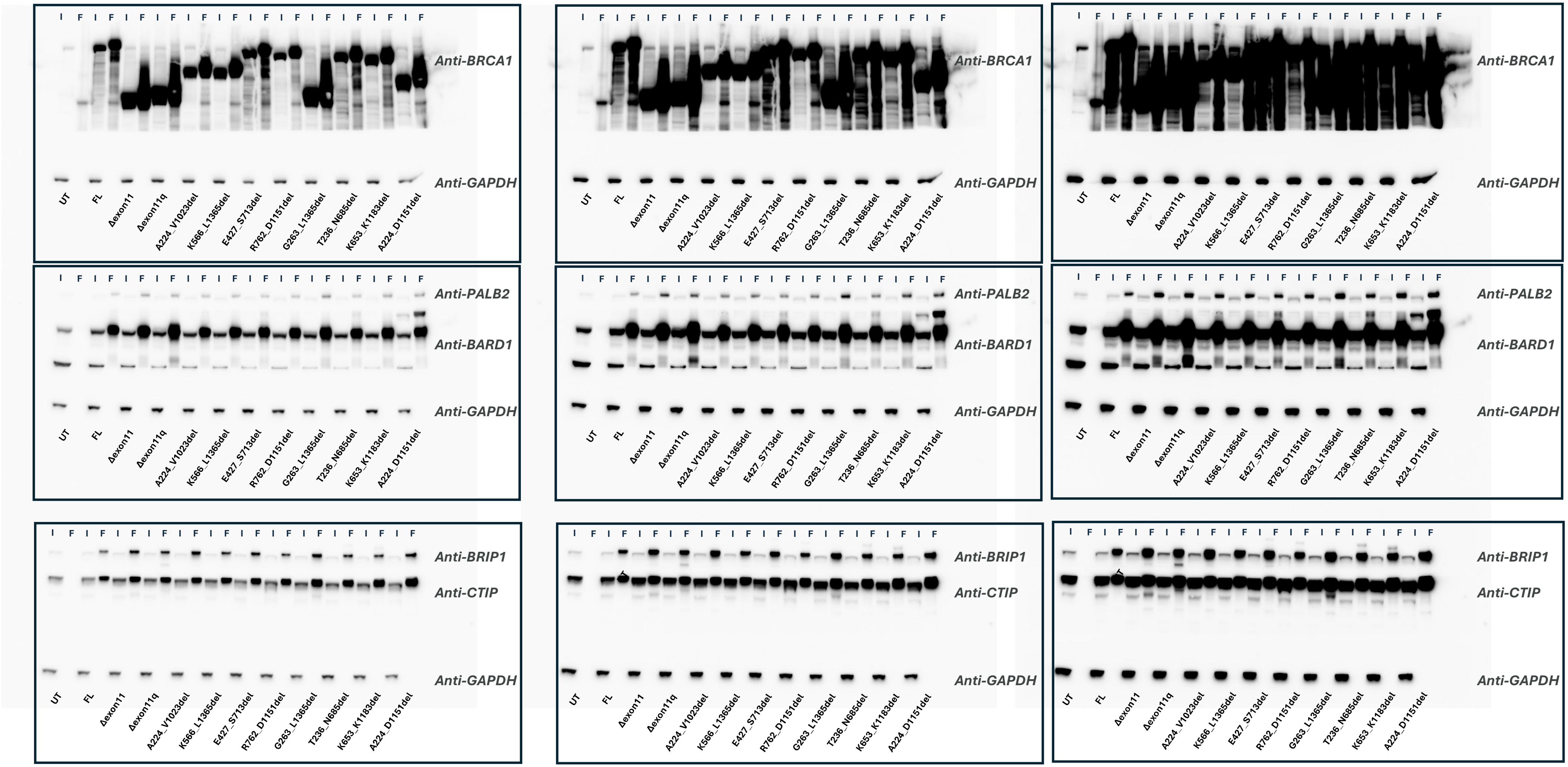

Figure S2 – B

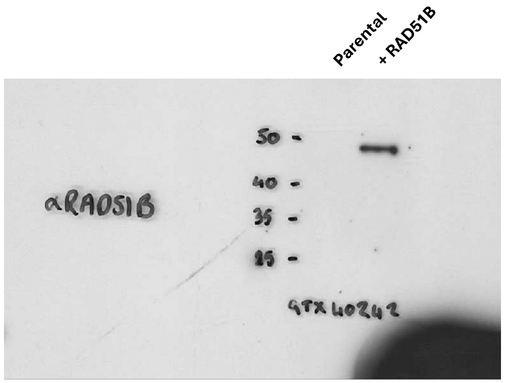

RAD51B

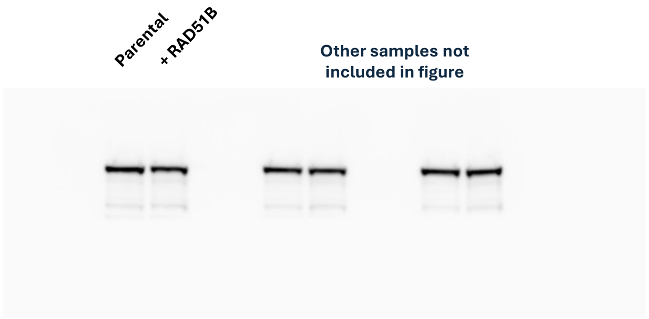

Vinculin

Figure S2 – C

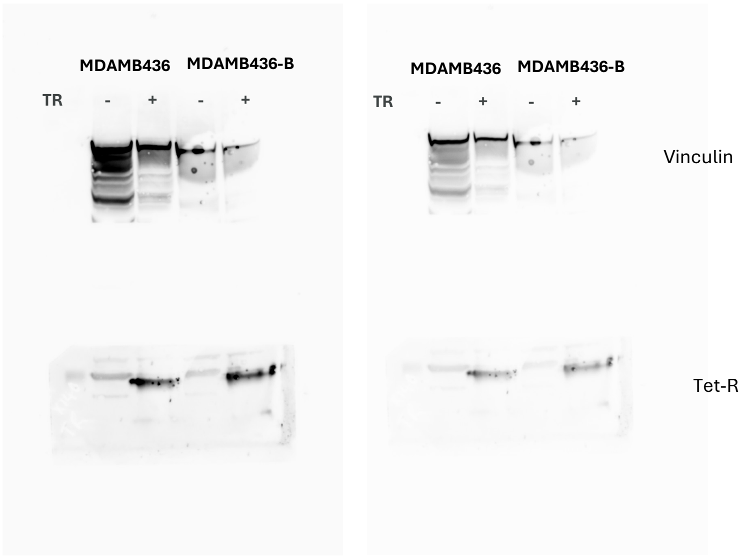
MDAMB436
MDAMB436-B
TR
-
+
-
+
MDAMB436
MDAMB436-B
TR
-
+
-
+
Vinculin
Tet-R

Figure S2 – D

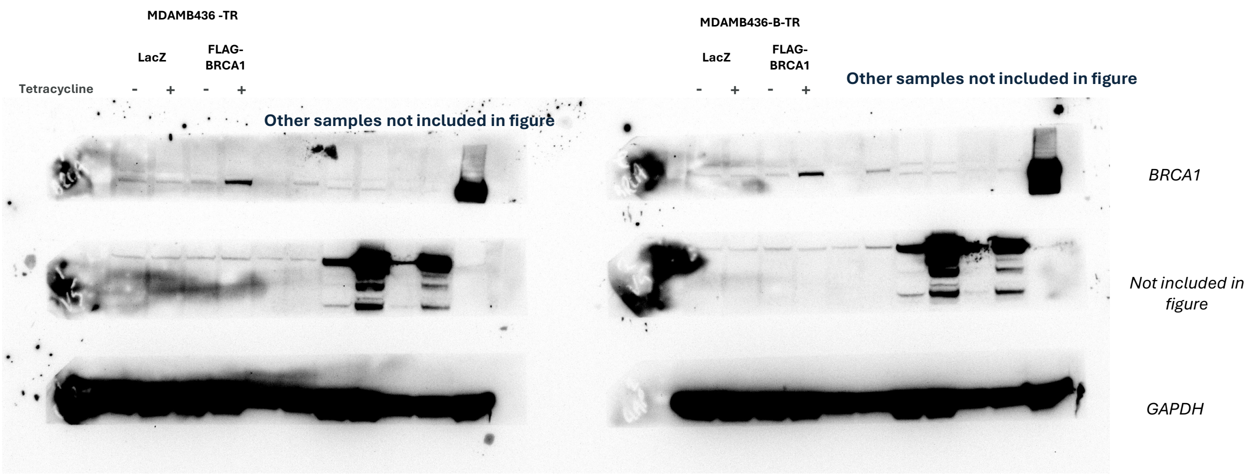
MDAMB436 -TR
LacZ
FLAG-BRCA1
Tetracycline
-
+
-
+
Other samples not included in figure
MDAMB436-B-TR
LacZ
FLAG-BRCA1
-
+
-
+
Other samples not included in figure
BRCA1
Not included in figure
GAPDH

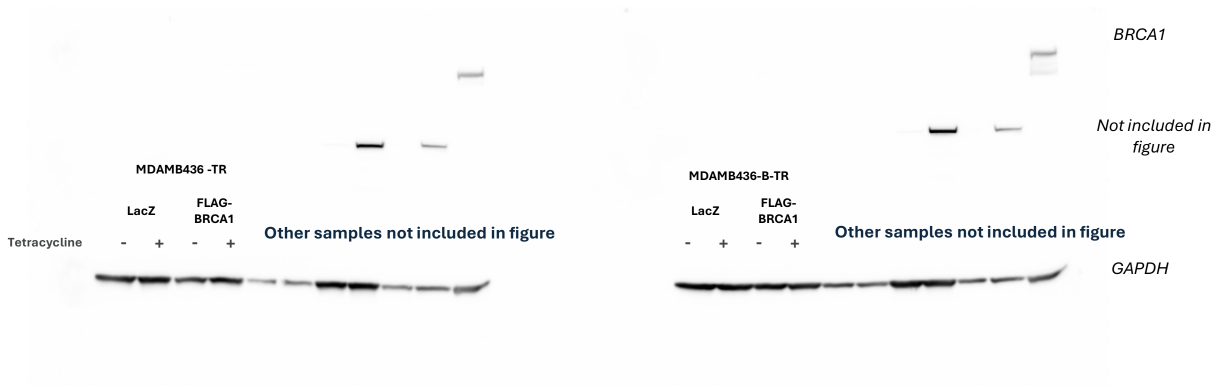
BRCA1
Not included in figure
MDAMB436 -TR
LacZ
FLAG-BRCA1
Tetracycline
-
+
-
+
Other samples not included in figure
MDAMB436-B-TR
LacZ
FLAG-BRCA1
-
+
-
+
Other samples not included in figure
GAPDH

Figure S2 – F

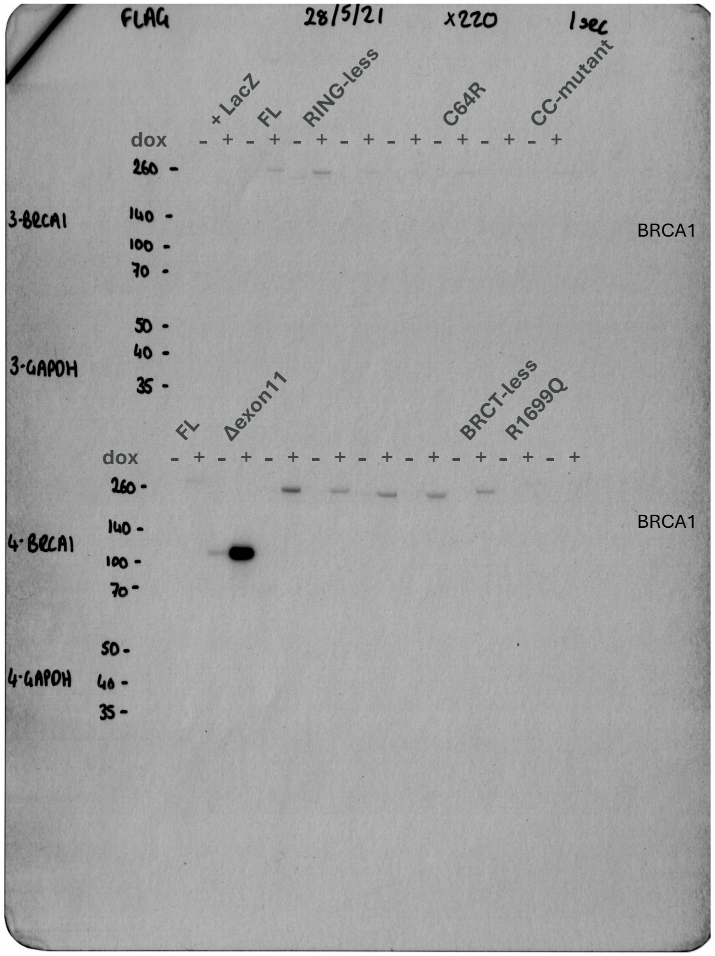
FLAG
28/5/21
x220
1sec
+LacZ
FL
RING-less
C64R
CC-mutant
dox
260
140
100
70
50
40
35
3-BRCA1
3-GAPDH
BRCA1
FL
Δexon11
BRCT-less
R1699Q
4-BRCA1
4-GAPDH

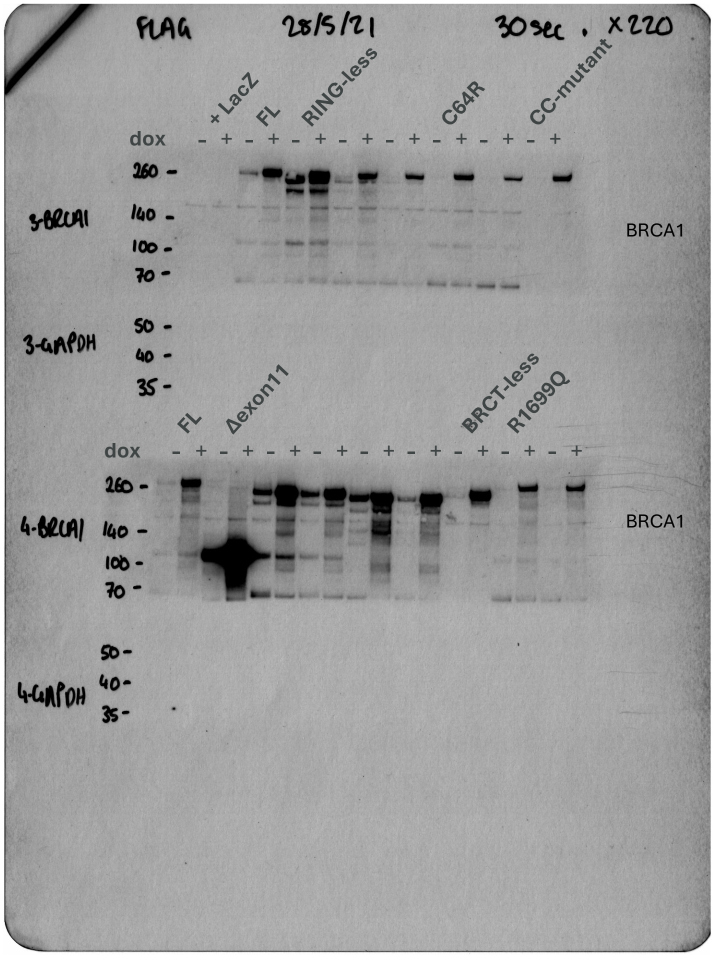
FLAG
28/5/21
30sec
x220
+LacZ
FL
RING-less
C64R
CC-mutant
dox
260
140
100
70
50
40
35
3-BRCA1
3-GAPDH
BRCA1
FL
Δexon11
BRCT-less
R1699Q
4-BRCA1
4-GAPDH

Figure S2 – F

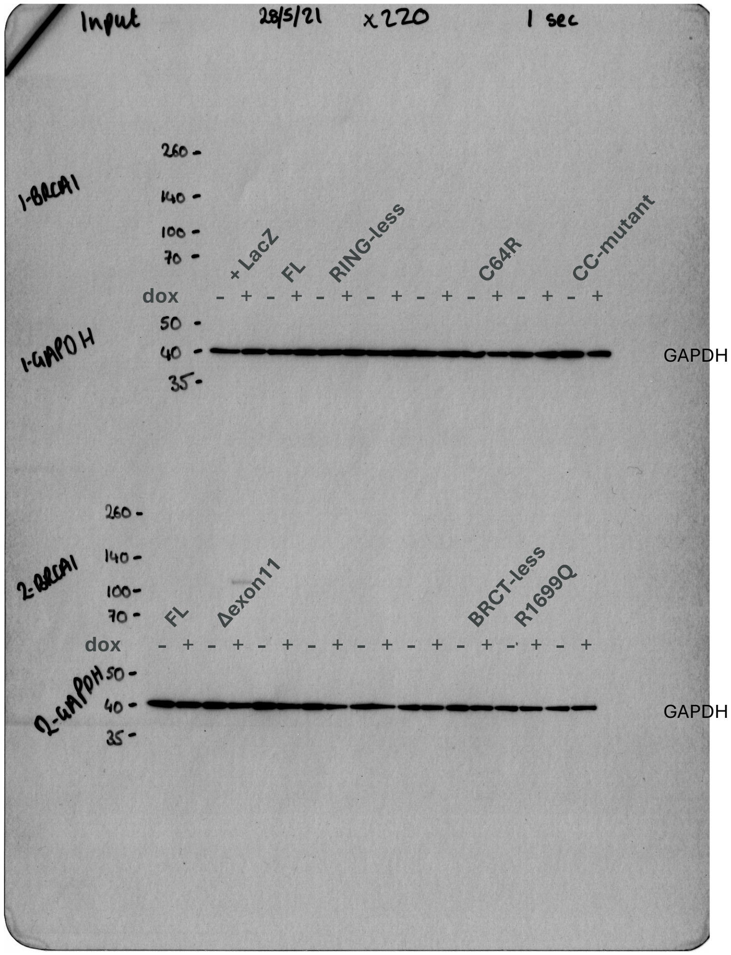

Input
28/5/21
x220
1 sec
1-BRCA1
260
140
100
70
+LacZ
FL
RING-less
C64R
CC-mutant
dox
- + - + - + - + - + - + - + - +
50
40
35
1-GAPDH
GAPDH
2-BRCA1
260
140
100
70
FL
Δexon11
BRCT-less
R1699Q
dox
- + - + - + - + - + - + - + - + - +
50
40
35
2-GAPDH
GAPDH
