# Supplementary material for: An Inducible BRCA1 Expression System with In Vivo Applicability Uncovers Activity of the Combination of ATR and PARP Inhibitors to Overcome Therapy Resistance
Source: Cancers (Basel). 2026 Jan 20;18(2):309. doi: 10.3390/cancers18020309 (PMC12838977; doi:10.3390/cancers18020309)
Supplement: Supplementary file 1 [file cancers-18-00309-s001.zip › cancers-4069969-supplementary/cancers-4069969-supplementary figures.pdf]

Supplementary figures

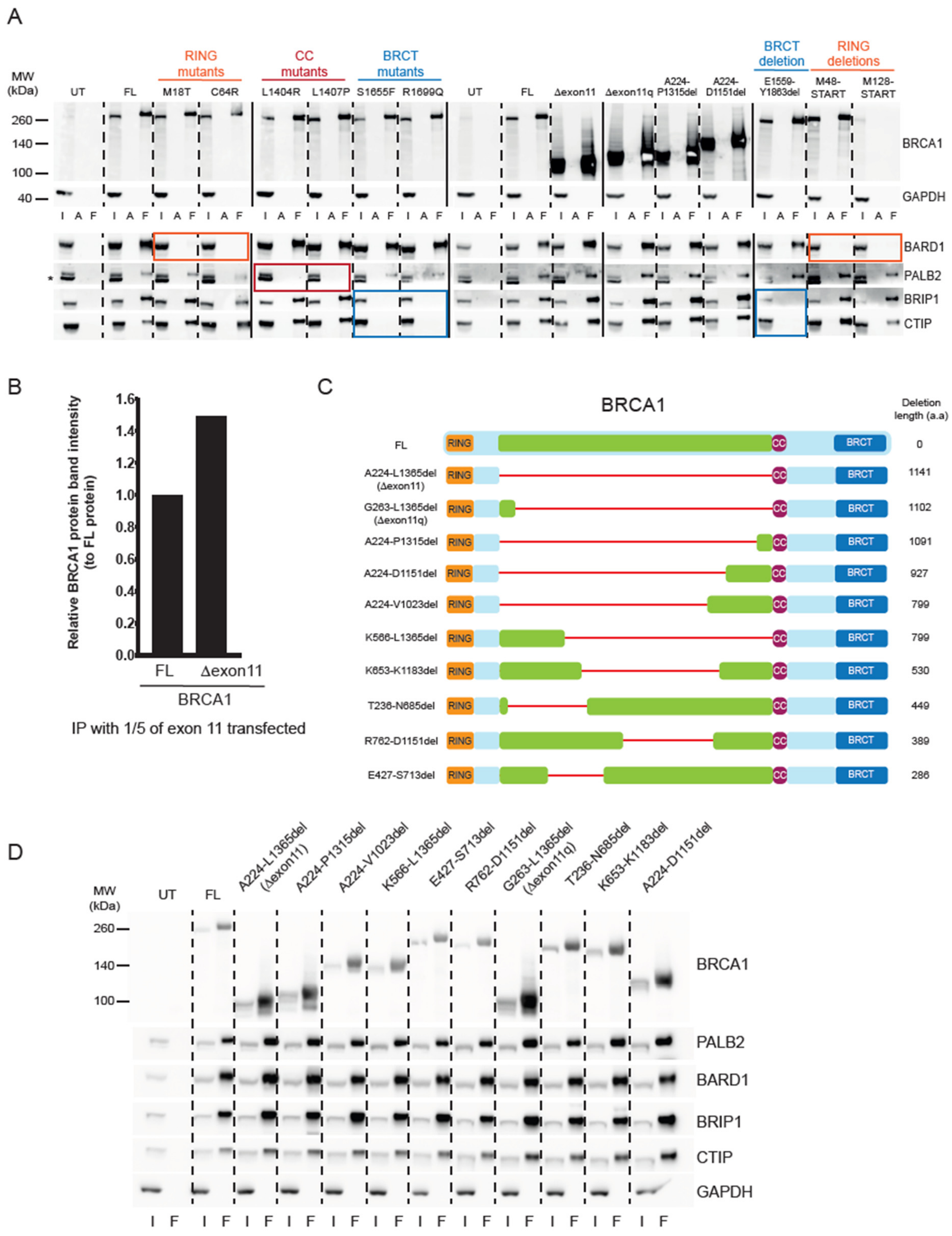

Supplementary Figure S1. (A) Co-immunoprecipitation experiments in HEK293T cells with the different forms of BRCA1 used in this study. UT: unstransfected; FL: full length; **I**: input; A: pull down with protein A beads; F: pull down with FLAG beads. **(B)** Quantification of the relative BRCA1

protein pull down normalized to the BRCA1 full length (FL) protein[illegible] related to Figure 1D. **(C)** Schematic of the BRCA1 exon 11 protein deletions analysed in this study. **(D)** Co-immunoprecipitation experiments in HEK293T cells with the different exon 11 deletions of BRCA1 used in this study. UT: unstransfected; FL: full length; **I**: input; F: pull down with FLAG beads.

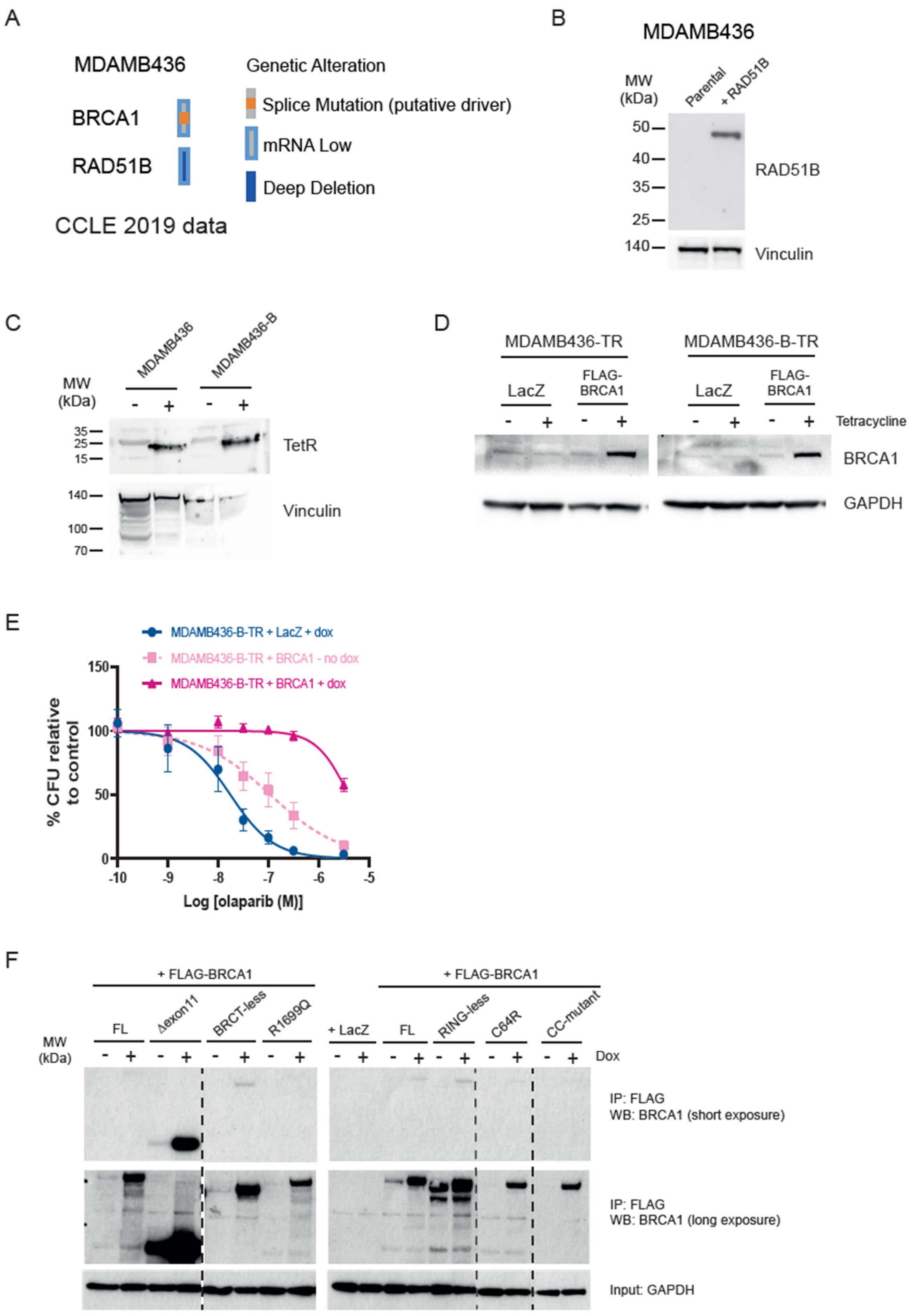

Supplementary Figure S2. (A) Genetic alterations in the MDAMB436 cell line as described in the Cancer Cell Line Encyclopaedia data update of 2019. **(B)** Western blot showing expression of RAD51B only in MDAMB436 cells stably transfected with a RAD51B expression construct.

Vinculin was used as loading control. **(C)** Western blot showing expression of the tetracycline repressor (TetR) only in MDAMB436 or MDAMB436-B (+RAD51B) cells transfected with the TetR construct (+). Vinculin was used as loading control. **(D)** Western blot showing expression of FLAG-tagged BRCA1 in the presence of tetracycline induction in MDAMB436-TR (+TetR) or MDAMB436-B-TR (+RAD51B +TetR) cells. GAPDH was used as loading control. **(E)** Dose-response curves of olaparib in colony formation assays in tetracycline-repressor (TR) expressing MDAMB436-B (+RAD51B) cells with (+BRCA1) or without (+LacZ) BRCA1 complementation cultured in the presence or absence of doxycycline (+/− dox). **(F)** Western blot showing immunoprecipitation of FLAG-tagged BRCA1 constructs (or LacZ control) stably transfected in MDAMB436-B-TR cells in the presence or absence of doxycycline induction. FL: full length. GAPDH was used as input control.
